# Associations of ^18^F‐RO‐948 tau PET with fluid AD biomarkers, Centiloid, and cognition in early AD continuum

**DOI:** 10.1002/alz.71176

**Published:** 2026-04-17

**Authors:** Mahnaz Shekari, Armand González Escalante, Marta Milà‐Alomà, Carles Falcon, David López‐Martos, Gonzalo Sánchez‐Benavides, Anna Brugulat‐Serrat, Aida Niñerola‐Baizán, Nicholas J. Ashton, Thomas K. Karikari, Juan Lantero‐Rodriguez, Laia Montoliu‐Gaya, Anniina Snellman, Theresa A. Day, Jeffrey L. Dage, Paula Ortiz‐Romero, Matteo Tonietto, Edilio Borroni, Gregory Klein, Gwendlyn Kollmorgen, Margherita Carboni, Clara Quijano‐Rubio, Eugeen Vanmechelen, Carolina Minguillón, Karine Fauria, Andrés Perissinotti, José Luis Molinuevo, Henrik Zetterberg, Kaj Blennow, Oriol Grau‐Rivera, Marc Suárez‐Calvet, Juan Domingo Gispert, for the ALFA Study

**Affiliations:** ^1^ Barcelonaβeta Brain Research Center (BBRC) Pasqual Maragall Foundation Barcelona Spain; ^2^ Hospital del Mar Research Institute (HMRI) Barcelona Spain; ^3^ Universitat Pompeu Fabra Barcelona Spain; ^4^ Centro de Investigación Biomédica en Red de Fragilidad y Envejecimiento Saludable (CIBERFES) Instituto de Salud Carlos III Madrid Spain; ^5^ Centro de Investigación Biomédica en Red de Bioingeniería, Biomateriales y Nanomedicina; (CIBERBBN) Instituto de Salud Carlos III Madrid Spain; ^6^ Global Brain Health Institute San Francisco California USA; ^7^ Nuclear Medicine Department Hospital Clínic Barcelona ‐ IDIBAPS Barcelona Spain; ^8^ F. Hoffmann‐La Roche Ltd Basel Switzerland; ^9^ Department of Psychiatry and Neurochemistry Institute of Neuroscience & Physiology the Sahlgrenska Academy at the University of Gothenburg Mölndal Sweden; ^10^ Banner Alzheimer's Institute and University of Arizona Phoenix Arizona USA; ^11^ Banner Sun Health Research Institute Sun City Arizona USA; ^12^ Department of Psychiatry University of Pittsburgh Pittsburgh Pennsylvania USA; ^13^ Turku PET Centre University of Turku Turku Finland; ^14^ Lilly Research Laboratories Eli Lilly and Company Indianapolis Indiana USA; ^15^ Stark Neurosciences Research Institute Indiana University School of Medicine Indianapolis Indiana USA; ^16^ Roche Diagnostics GmbH Penzberg Germany; ^17^ Roche Diagnostics International Ltd Rotkreuz Switzerland; ^18^ ADx NeuroSciences Ghent Belgium; ^19^ Novartis Institutes for BioMedical Research Translational Medicine, Neuroscience Basel Switzerland; ^20^ Department of Psychiatry and Neurochemistry Institute of Neuroscience and Physiology University of Gothenburg Mölndal Sweden; ^21^ Clinical Neurochemistry Laboratory Sahlgrenska University Hospital Mölndal Sweden; ^22^ Department of Neurodegenerative Disease UCL Institute of Neurology London UK; ^23^ UK Dementia Research Institute at UCL London UK; ^24^ Servei de Neurologia Hospital del Mar Barcelona Spain

**Keywords:** 18 < /u > f‐ro‐948 pet, Alzheimer's disease, fluid biomarkers, neurofibrillary tangle (NFT), tau pathology

## Abstract

**INTRODUCTION:**

We examined neurofibrillary tangle (NFT) pathology using 18F‐RO‐948 tau positron emission tomography (PET) in cognitively unimpaired individuals and its associations with amyloid plaques, fluid biomarkers, and cognition in early preclinical Alzheimer's disease (AD).

**METHODS:**

We analyzed 97 participants from the ALFA+ cohort with tau and amyloid PET, magnetic resonance imaging, fluid biomarkers (cerebrospinal fluid [CSF]/plasma), and cognitive data. Braak staging was applied, and correlations with biomarkers and cognitive measures were assessed. Receiver operating characteristic analyses evaluated biomarker performance in predicting tau PET positivity

**RESULTS:**

CSF and plasma tau phosphorylated at threonine 217 (p‐tau217) showed the strongest correlation with early Braak I/II tau PET signal (*r* = 0.58, and *r* = 0.37, respectively), while plasma p‐tau181 and p‐tau181/Aβ42 showed moderate associations (*r* ∼ 0.25). However, positive predictive values were low (PPV = 0.09–0.33).

**DISCUSSION:**

^18^F‐RO‐948 PET detected early tau pathology in individuals with low to moderate amyloid load. Fluid biomarkers, especially in plasma, had limited predictive power but high negative predictive value, supporting their use in ruling out early tau pathology in preclinical AD.

## Highlights

- Abnormal ^1^ ^8^F‐RO‐948 uptake could be detected in CU individuals. 
- Tau PET signal abnormalities observed even in subjects with low amyloid burden.
- Most tau‐positive cases showed elevated Braak I/II uptake, with notable exceptions.
- CSF/plasma p‐tau217 and p‐tau181/Aβ42 ratios exhibited the largest fold changes across AT stages. 
- Fluid biomarkers' high NPV (>94%) supports potential screening applications.

## 1 BACKGROUND

Neurofibrillary tangles (NFTs), composed of hyperphosphorylated tau, are the defining characteristic of Alzheimer's disease (AD), together with fibrillar amyloid beta (Aβ) deposits in the brain.^1^, ^2^, ^3^, ^4^ Typically, NFTs appear later than Aβ in the preclinical stages of the disease and is a strong predictor of imminent neurodegeneration^5^ and cognitive decline. Several radiotracers have shown their capacity to image in vivo the amount and spread of NFT pathology.^6^ These tracers have been validated against neuropathology,^7^ and some have been or are being developed for diagnostic clinical use.^8^ Several tau positron emission tomography (PET) radiotracers, including ^1^ ^8^F‐flortaucipir and ^1^ ^8^F‐MK‐6240, have been validated through *post mortem* studies, showing strong concordance with the distribution and burden of tau pathology in the brain.^9^ Furthermore, head‐to‐head comparisons among ^1^ ^8^F‐flortaucipir, ^1^ ^8^F‐RO‐948, and ^1^ ^8^F‐MK‐6240 have demonstrated high inter‐tracer agreement in tau PET signal patterns,^10^, ^11^ reinforcing their reliability in capturing tau pathology in vivo and supporting their validity against neuropathological standards.

The spread of NFTs in AD is typically believed to follow neuropathologically defined Braak staging.^4^, ^12^ According to the prototypical Braak spread pattern, tau starts accumulating in medial temporal regions (Braak I/II) before spreading to limbic regions (Braak III/IV) and finally to the whole cortical mantle (Braak V/VI). This pattern of cortical spread has been formalized into the hierarchical Braak staging system, which is part of the gold‐standard diagnostic workup for AD at autopsy.^3^ However, recent in vivo tau PET studies across multiple cohorts suggest that several subtypes of tau spread can be identified with distinct demographic characteristics, cognitive profiles, and longitudinal outcomes.^13^, ^14^ This highlights the added value of tau PET for the study of the pathological diversity of AD and its relationship with the observed heterogeneity in clinical symptoms.^15^

While tau PET imaging can accurately detect and quantify NFT pathology, there is an urgent need for inexpensive and minimally invasive blood AD biomarkers to detect early disease changes. Efficient blood‐based biomarkers may also guide treatment decisions and support clinical management in practice. In recent years, several highly specific and potentially useful blood‐based biomarkers for AD have been identified,^16^ with phosphorylated tau (p‐tau) variants among the most promising. Several studies have shown that tau phosphorylated at threonine 181 (p‐tau181), p‐tau217, and p‐tau231 are highly specific for AD and rise early on the preclinical AD continuum.^17^, ^18^ However, neuropathology studies show that plasma p‐tau181 and p‐tau217 mark both Aβ and NFT pathology, whereas plasma p‐tau231 is mainly associated with Aβ.^19^, ^20^ Recent reports on cerebrospinal fluid (CSF) biomarkers, particularly CSF p‐tau205^21^ and CSF MTBR‐tau243 (microtubule‐binding region of tau containing the residue 243),^22^ suggest that they are more specific to aggregated insoluble tau pathology; however, their blood‐based counterparts await full characterization and validation.

In summary, while blood‐based biomarkers’ capacity to predict the presence of Aβ pathology using amyloid PET is good,^23^, ^24^, ^25^ their concordance with tau PET remains low. This is further aggravated in the preclinical stages of AD, where changes in NFTs are subtle compared to more advanced stages.^26^ Therefore, there is a need for blood‐based biomarkers that can specifically inform on the presence of tau pathology in preclinical AD. Additionally, there is limited evidence in the literature reporting the extent to which different regional patterns of tau pathology can be detected in preclinical stages of AD.

In this work, we aimed to assess NFT pathology in the early preclinical AD continuum, as assessed by 18F‐RO‐948 tau PET imaging, and its relationship with other AD biomarkers. To this end, we acquired Aβ and tau PET, quantified several tau phospho‐forms in the CBF and blood, and assessed their agreement in a sample of the ALFA+ cohort of cognitively unimpaired (CU) participants selected based on their baseline levels of Aβ load.^27^

## 2 METHODS

### 2.1 Participants

A total of 100 CU participants with available amyloid PET scans, tau PET scans, T1‐weighted magnetic resonance imaging (MRI), fluid AD biomarkers, and cognition scores were selected from the ALFA+ cohort.^27^ These participants were selected based on CSF‐derived AT staging, where “A” indicates amyloid positivity (defined by the CSF Aβ42/40 ratio cut‐off of 0.071) and “T” indicates tau positivity (defined by the CSF p‐tau181 cut‐off of 24 pg/mL), with the aim being the most balanced distribution across the four groups (A−T−, A+T−, A+T+, and A−T+).^28^ More information about the tau PET study design can be found at https://clinicaltrials.gov/study/NCT04482660. ALFA+ is a longitudinal study of 450 CU individuals enriched for a family history of AD and carriership of the ε4 variant of the apolipoprotein E (*APOE ε4)* gene. Inclusion and exclusion criteria, along with study details, have been previously described.^27^

All participants gave written informed consent. The ALFA+ study was approved by the independent ethics committee “Parc de Salut Mar,” Barcelona, and is registered at Clinicaltrials.gov (Identifier: NCT02485730).

**RESEARCH IN CONTEXT**

1. **Systematic review**: We conducted a PubMed literature search on studies examining the relationship between fluid biomarkers and tau PET imaging in CU individuals. Existing research primarily includes individuals with more advanced AD pathology, such as a higher prevalence of amyloid positivity and older age, limiting insights into the earliest stages of tau deposition.
2. **Interpretation**: Our findings confirm that tau tangles are detectable by tau PET in the population at risk of AD. Additionally, we showed that, although all fluid biomarkers and Centiloid outperformed demographic (age, sex, *APOE ɛ4*) in predicting early tau PET positivity, their weak PPVs suggest they are inadequate for screening early tau pathology or selecting patients for early intervention clinical trials. However, their high negative predictive values highlight the utility of fluid biomarkers in excluding early tau pathology in the diagnostic context.
3. **Future directions**: While our study used a cohort enriched for AD pathology, larger sample sizes are needed to validate these findings. Additionally, longitudinal studies are essential to better characterize tau progression patterns and their associations with fluid biomarkers, providing deeper insight into the dynamics of tau pathology in CU individuals at risk of AD.

### 2.2 PET and MRI acquisition

Amyloid PET scans were conducted between March 6, 2020, and June 27, 2022, while tau PET scans were performed between March 29, 2021, and February 7, 2023. Aβ PET and tau PET data acquisition was performed for 20 min (4 frames × 5 min) on a Siemens Biograph mCT scanner, following a cranial computed tomography (CT) scan for attenuation correction. Tau PET images were acquired 70 to 90 min after injecting 368.75 ± 13.02 MBq of ^18^F‐RO‐948, and Aβ PET scans were collected 90 to 110 min after injecting ∼185 MBq of ^18^F‐flutemetamol. Both tau PET and Aβ PET scans were reconstructed using an ordered subset expectation maximization (OSEM) algorithm and incorporating time of flight (TOF) and point spread function modeling (tau PET: four iterations, 21 subsets, 4 mm; Aβ PET: eight iterations, 21 subsets, 3 mm).

T1‐weighted MRI scans were obtained using a 3T scanner (Ingenia CX, Philips Healthcare, Best, the Netherlands) with a 32‐channel head coil and a 3D turbo field echo sequence with the following parameters: echo time/repetition time = 4.6/9.9 ms, flip angle = 8°, and voxel size = 0.75 × 0.75 × 0.75 mm.

### 2.3 PET quantification

Prior to preprocessing, all PET scans underwent quality control. One tau PET scan was excluded because it was acquired significantly later than the recommended post‐injection time, making the data unreliable. Next, the images were processed with SPM12 (www.fil.ion.ucl.ac.uk/spm). Standardized uptake value ratio (SUVR) parametric images of ^18^F‐RO‐948 were created in the subject space by dividing the voxel intensities by the average intensity in a reference region covering the inferior cerebellum using the SUIT atlas.^29^, ^30^ For creating Braak regions of interest (ROIs), a composite atlas was created using the predefined ROIs defined elsewhere.^31^ To create a subject‐based Braak atlas, first the Braak atlas template was moved to the native space using the deformation field, extracted by segmenting the T1‐weighted MRI. Next, the Braak atlas template was coregistered and resliced with T1‐weighted MRI. Then a binary gray matter (GM) mask was created where the probabilities of GM > WM (white matter) and GM > CSF were assigned to 1, and the rest were assigned to zero. Finally, the Braak atlas template mask was multiplied by the binary GM mask to create a subject‐based Braak mask (Figure S1B). In addition to the subject‐based Braak mask, subject‐based meninges and skull masks were created by segmenting the corresponding CT scan of the participant (Figure S1A). Mean meningeal and skull uptakes were created for all participants. Pearson correlation between Braak ROI uptake and meningeal SUVR was calculated for all participants. A statistically significant correlation between any Braak ROI and meningeal uptake was interpreted as meningeal contamination of the cortical Braak ROI. To minimize this contamination, only individuals with meningeal SUVR > 2 were selected for cortical sharpening. The meningeal mask was then dilated one to 10 times for each participant with meningeal SUVR above 2. Voxels overlapping between the dilated meningeal mask and cortical ROIs were removed to refine the cortical mask. The cortical SUVR for each Braak stage was then calculated using this sharpened cortical mask. The optimal dilation level was determined when SUVR values became independent of further sharpening (Figures S2 and S3). As a final quality control step, the Pearson correlation between Braak ROIs and meningeal SUVR was recalculated, and the absence of a significant correlation was considered evidence of reliable quantification.

In addition to the PET‐based Braak ROIs, we defined a temporal MetaROI composite and a NEO‐T composite. Temporal MetaROI consisted of the amygdala, parahippocampal gyrus, fusiform gyrus, and the middle and inferior temporal gyri,^32^ whereas the NEO‐T composite included bilateral middle and inferior temporal gyri.

Finally, SUVRs were computed for cortical regions approximating Braak stages, entorhinal cortex (Braak I/II), limbic temporal (Braak III/IV), and neocortical association cortex (Braak V/VI), as well as for the temporal MetaROI and NEO‐T composites. Regional positivity for each Braak stage (Cut‐off‐Braak I/II, Cut‐off‐Braak III/IV, Cut‐off‐Braak V/VI), the temporal MetaROI, and NEO‐T was defined as mean plus two standard deviations (SDs) of SUVRs in the reference control group (CSF A−T−), calculated separately for each ROI/composite. Tau PET staging for each Braak ROI, the global region, temporal MetaROI, and NEO‐T was determined using the calculated thresholds. In addition to SUVR, all images were quantified using the CenTauR‐z method.^33^ CenTauR‐z was developed to standardize tau PET quantification across different tracers by generating *z*‐scored SUVR values for predefined cortical ROIs. The meta‐ROI template used for this analysis is available on the GAAIN website.^34^ Further details on the preprocessing steps can be found in the original publication.^33^

Aβ PET scans were quantified in Centiloid (CL) units, using a validated in‐house standard pipeline.^35^, ^36^ In brief, an average PET image was created after realigning four frames to correct for any possible motions between frames. Then the PET scans were coregistered with their corresponding T1‐weighted MRI scan and warped to Montreal Neurological Institute (MNI) space. The SUVR maps were created using the predefined GAAIN whole cerebellum mask as the reference region. Finally, SUVR was calculated for the GAAIN predefined cortical target region and transformed into the CL.^37^

### 2.4 Plasma and CSF biomarkers

Fluid biomarker samples were collected within 3.00 ± 4.08 months of the amyloid PET scan and 9.42 ± 9.31 months of tau PET scans. CSF and blood biomarkers were collected and processed using different assays, following standard procedures.^38^, ^39^ CSF p‐tau205, p‐tau235, and NTA‐tau (N‐terminal tau), as well as plasma p‐tau181, p‐tau231, and Aβ42 concentrations, were measured using either commercial or in‐house single‐plex assays on the Simoa HD‐X platform (Quanterix, Billerica, MA, USA). CSF p‐tau181, Aβ40, and Aβ42 were measured using the electrochemiluminescence Elecsys immunoassay on a fully automated cobas e601 module (both Roche Diagnostics International, Rotkreuz, Switzerland). CSF (p‐tau181, Aβ40, Aβ42, t‐tau) and plasma (Aβ40, Aβ42, p‐tau181) were measured using the Roche NeuroToolKit immunoassays (Roche Diagnostics International) on a cobas e411 or e601 analyzer. Additionally, p‐tau217 in both CSF and plasma was measured using an Eli Lilly assay on the Meso Scale Discovery (MSD) platform.^40^ All participants were categorized in AT stages according to CSF using pre‐established CSF‐based cut‐off values (CSF Aβ42/40 < 0.071 [A+] and CSF p‐tau181 > 24 pg/mL [T+]).^28^

### 2.5 Neuropsychological evaluation

A modified version of the Preclinical Alzheimer's Cognitive Composite (PACC) score, consisting of attention, executive, language, memory, and visual composites, was available for all participants.^41^, ^42^, ^43^ All raw test scores were standardized into *z*‐scores using the mean and SD from CU CSF A−T− participants as a reference and then averaged into a composite score.^43^

### 2.6 Statistical analyses

Descriptive statistics were calculated for the main demographic variables of the participants grouped by the CSF‐based AT status. Correlations between Centiloid, ^18^F‐RO‐948 SUVRs in each Braak region, temporal MetaROI, and NEO‐T and AD biomarkers were assessed using partial correlation, adjusted for age, sex, *APOE ɛ4* carriership, and the time interval between PET imaging and fluid biomarker collection (∆Time).

(1) TauSUVR/Centiloid∼1+Biomarker+Age+Sex+APOEε4+ΔTime

To compare the strength of correlations between modalities and regions (Centiloid vs Braak, Centiloid vs MetaROI, Braak vs MetaROI), we applied Williams’ test. For each comparison, the difference in correlation coefficients (Δ*r*) and corresponding *p* values are reported. Multiple comparisons were corrected using the Benjamini–Hochberg FDR procedure, with *q* < 0.05 considered significant.

Receiver operating characteristic (ROC) analyses were performed to assess the ability of fluid biomarkers to predict Braak I/II positivity in CU individuals. Analyses were performed using models that included biomarkers alone as well as models adjusted for age, sex, and *APOE ɛ4* carriership. Youden's Index (YI) was used to determine optimal thresholds using the results of the adjusted model. In particular, the optimal CL cut‐off to predict Braak I/II positivity (Cut‐off‐Centiloid‐Braak I/II) was also calculated. AUC values of [0.7 to 0.8] were considered acceptable, [0.8 to 0.9] as excellent, and above 0.9 as outstanding performance in discrimination. Additionally, the positive predictive value (PPV) and negative predictive value (NPV) of each biomarker were calculated.

A posteriori, we defined four PET‐based AT stages based on amyloid PET (A) and tau PET (T) positivity.^44^ Under this scheme, participants were classified as follows:

- PET (A−T−): Centiloid < 12 & Braak I/II < Cut‐off‐Braak I/II
- PET (A(Gz)T−): 12 ≤ Centiloid < Cut‐off‐Centiloid‐Braak I/II and Braak I/II < Cut‐off‐Braak I/II
- PET(A+T−): Centiloid ≥ Cut‐off‐Centiloid‐Braak I/II and Braak I/II < Cut‐off‐Braak I/II
- PET (A+T+): Centiloid ≥ Cut‐off‐Centiloid‐Braak I/II and Braak I/II ≥ Cut‐off‐Braak I/II

On top of the data‐driven cut‐offs for CL and tau PET positivity, we also applied a CL < 12 threshold based on prior literature supporting its sensitivity for detecting early amyloid abnormalities.^45^ All biomarkers were *z*‐scored using the CSF‐based A−T− group as the reference for standardized comparisons. To examine group differences, we used the Kruskal–Wallis test across the four PET‐derived AT groups and applied Bonferroni correction for multiple comparisons.

To assess the magnitude of biomarker changes across PET‐defined disease stages, we additionally calculated fold changes using the raw (non‐*z*‐scored) fluid biomarker values. Each value was divided by the mean of the corresponding biomarker in the PET A−T− reference group (individuals who were negative for both amyloid and tau PET). These fold changes offer a complementary view of absolute biomarker shifts associated with disease progression. For all analyses, *p* values < 0.05 were considered statistically significant.

## 3 RESULTS

### 3.1 Demographic information and staging

Table 1 presents demographic characteristics, CL values, hippocampal volumes, regional tau PET SUVRs across Braak stages, temporal MetaROI, NEO‐T, and CenTauR‐z scores, and PACC scores for participants with available tau and amyloid PET scans after quality control, stratified by CSF‐based AT status.

**TABLE 1:** Demographic characteristics, Centiloid values, and PACC scores of participants, stratified by CSF‐based amyloid (A) and tau (T) status. Biomarker samples were collected within 9.42 ± 9.31 months of tau PET imaging. Participants were classified into AT stages using pre‐established cut‐offs based on CSF Aβ42/40 < 0.071 and p‐tau181 > 24 (pg/mL) measured with the NeuroToolKit, a panel of robust prototype assays and Elecsys immunoassays (Roche Diagnostics International Ltd). Between‐group comparisons were performed using ANOVA, followed by Bonferroni post hoc corrections, with A−T− as the reference group. Statistical significance (*p* < 0.001) for multiple comparisons is indicated by “**”. It should be noted that 2 of individuals did not have AT status due to lack of available CSF data and not included in the table.

	Total	A−T−	A+T−	A+T+	A−T+
*N*	97	26	33	30	8
*Age, mean (SD) [53 to 78 years]*	65.15 ± 5.00	64.23 ± 5.07	65.26 ± 4.87	66.13 ± 5.48	64.00 ± 3.16
*Sex, N (%) [Female]*	42 (42.42%)	13 (50.00%)	14 (42.42%)	9 (30.00%)	5 (62.50%)
*APOE ε4, N (%) [carriers]*	63 (63.64%)	11 (42.31%)	27 (81.81%)*	20 (66.67%)	3 (37.50%)
*Years of education*	13.35 ± 3.78	14.14 ± 3.86	14.12 ± 3.48	11.92 ± 3.71	13.77 ± 3.52
*CSF A*β*42/40, mean (SD)*	0.07 ± 0.03	0.09 ± 0.01	0.05 ± 0.01	0.04 ± 0.01	0.12 ± 0.02
*CSF p‐tau181, mean (SD), pg/mL*	21.92 ± 9.40	15.05 ± 4.55	17.99 ± 4.73	31.08 ± 9.51**	27.56 ± 5.00**
*CSF p‐tau181/A*β*40*	(1.06 ± 0.3) × 10^−3^	(0.82 ± 0.12) × 10^−3^	(0.98 ± 0.19) × 10^−3^	(1.40 ± 0.3) × 10^−3^	(0.97 ± 0.10) × 10^−3^
*CSF p‐tau181/A*β*42*	0.02 ± 0.01	0.009 ± 0.02	0.02 ± 0.007	0.03 ± 0.01	0.008 ± 0.001
*Centiloid*	22.51 ± 26.25	1.20 ± 8.35**	23.38 ± 21.43**	48.45 ± 21.75	−6.72 ± 7.45
*Braak I/II SUVR*	1.15 ± 0.19	1.07 ± 0.14	1.16 ± 0.14*	1.22 ± 0.26	1.10 ± 0.09
*Braak III/IV SUVR*	1.16 ± 0.12	1.11 ± 0.10	1.15 ± 0.08	1.21 ± 0.17	1.15 ± 0.02
*Braak V/VI SUVR*	1.08 ± 0.08	1.06 ± 0.09	1.09 ± 0.07	1.11 ± 0.09	1.08 ± 0.04
*Global tau PET SUVR*	1.11 ± 0.09	1.08 ± 0.09	1.11 ± 0.07	1.14 ± 0.11	1.10 ± 0.03
*Temporal MetaROI*	1.22 ± 0.16	1.15 ± 0.11	1.21 ± 0.10	1.29 ± 0.24	1.19 ± 0.03
*NEO‐T composite*	1.19 ± 0.12	1.14 ± 0.10	1.18 ± 0.09	1.24 ± 0.16	1.18 ± 0.02
*CenTauR‐z MetaROI*	1.09 ± 2.66	0.06 ± 1.67	0.93 ± 1.76	2.37 ± 3.86	0.39 ± 0.72
*Hippocampal volume (mm^3^)*	8860.14 ± 1521.54	8566.00 ± 1130.95	8670.81 ± 831.13	8380.19 ± 1219.52	9245.44 ± 556.99
*Z‐scored PACC, mean (SD)*	0.06 ± 0.63	0.19 ± 0.60	0.09 ± 0.57	−0.17 ± 0.69	0.30 ± 0.59

*Note*: Biomarker samples were collected within 9.42 ± 9.31 months of tau PET imaging. Participants were classified into AT stages using pre‐established cut‐offs based on CSF Aβ42/40 < 0.071 and p‐tau181 > 24 (pg/mL) measured with the NeuroToolKit, a panel of robust prototype assays and Elecsys immunoassays (Roche Diagnostics International Ltd). Between‐group comparisons were performed using ANOVA, followed by Bonferroni post hoc corrections, with A−T− as the reference group. Statistical significance (*p* < 0.001) for multiple comparisons is indicated by “**”. It should be noted that two individuals did not have AT status due to a lack of available CSF data and not included in the table.

Abbreviations: Aβ, amyloid beta; *APOE*, apolipoprotein E; CSF, cerebrospinal fluid; PACC, Preclinical Alzheimer's Cognitive Composite; PET, positron emission tomography; p‐tau, phosphorylated tau; ROI, region of interest; SD, standard deviation; SUVR, standardized uptake value ratio;

Based on the positivity threshold for each Braak stage, nine cases (9.09%) were positive in the Braak I/II region. Following a hierarchical pattern, five of them (5.05%) were also positive for the Braak III/IV ROI and one for the Braak V/VI region (Table S1). However, two scans did not follow the hierarchical pattern and were positive only in the Braak III/IV ROI (Figure S4). Both participants were in their 70s, *APOE ɛ4* carriers, female, with a high level of CL (89 and 78), and 8 years of education. Visual inspection of both scans confirmed an elevated level of tau PET signals in the Braak III/IV region, reassuring correct staging (Figure S5). Using the temporal MetaROI and NEO‐T composite, seven (7.07%) and six (6.06%) participants were positive, with overlapping among those classified as Braak III/IV positive.

**FIGURE 1:**
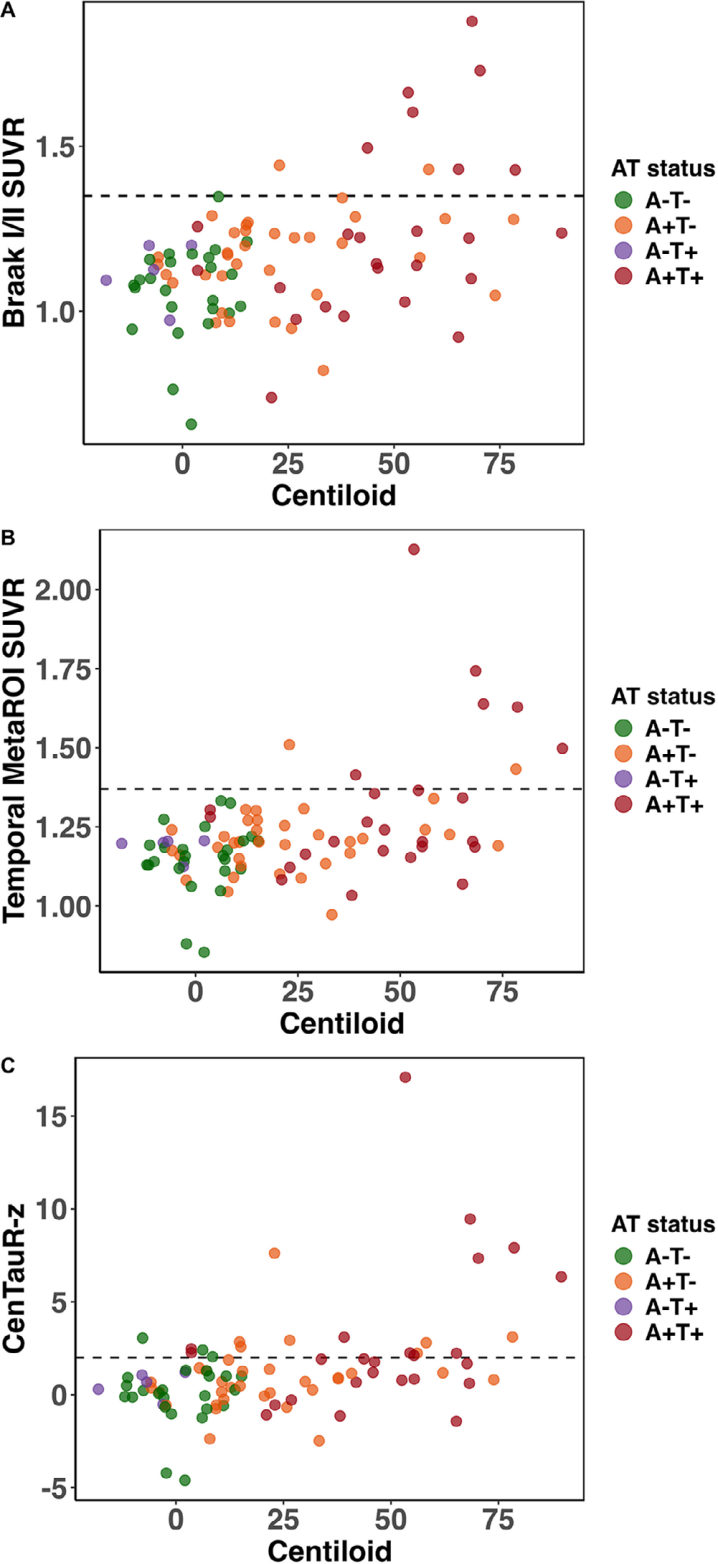
Scatter plot of Centiloid values (*x*‐axis) and (A) Braak I/II tau PET SUVR (*y*‐axis), (B) temporal MetaROI, (C) CenTauR‐z (MetaROI) with data points color‐coded by CSF‐based AT status: A−T− (green), A+T− (orange), A+T+ (red), A−T+ (purple). The dotted horizontal line represents the predefined standardized uptake value ratio (SUVR) threshold for tau positivity in Braak I/II = 1.35, temporal MetaROI = 1.37, and *z* = 2 for CenTauR‐z, respectively. Higher Centiloid values are associated with increased tau PET SUVR, particularly in the A+T+ group (red), indicating a relationship between amyloid burden and early tau deposition in the Braak I/II region and temporal MetaROI.

In addition to SUVR, CenTauR‐z in MetaROI was calculated for all tau PET scans. Figure 1 illustrates the relationship between Centiloid values and Braak I/II SUVR, temporal MetaROI, and CenTauR‐z, stratified by CSF‐based AT status. CenTauR‐z and Braak I/II SUVR have a correlation of *r* = 0.82 (95% CI: [0.60 to 0.92], *p* < 0.001). The correlation between CL and both Braak I/II SUVR and temporal MetaROI SUVR was statistically significant only in the A+T+ group (Braak I/II: *r* = 0.53, 95% CI: 0.10 to 0.83, *p* = 0.01; temporal MetaROI: *r* = 0.46, 95% CI: 0.04 to 0.75, *p* = 0.03). Using a linear regression model, CenTauR‐z = 2 was equivalent to 1.19 Braak I/II SUVR (Braak I/II = 1.09 + 0.05 × CenTauR‐z) and 1.26 temporal MetaROI SUVR (temporal MetaROI = 1.15 + 0.059× CenTauR‐z), which is lower than the estimated positivity thresholds for both ROIs and resulted in a higher number of tau PET scans with CenTauR‐z above 2.

### 3.2 Correlations with biofluids and Centiloids

**FIGURE 2:**
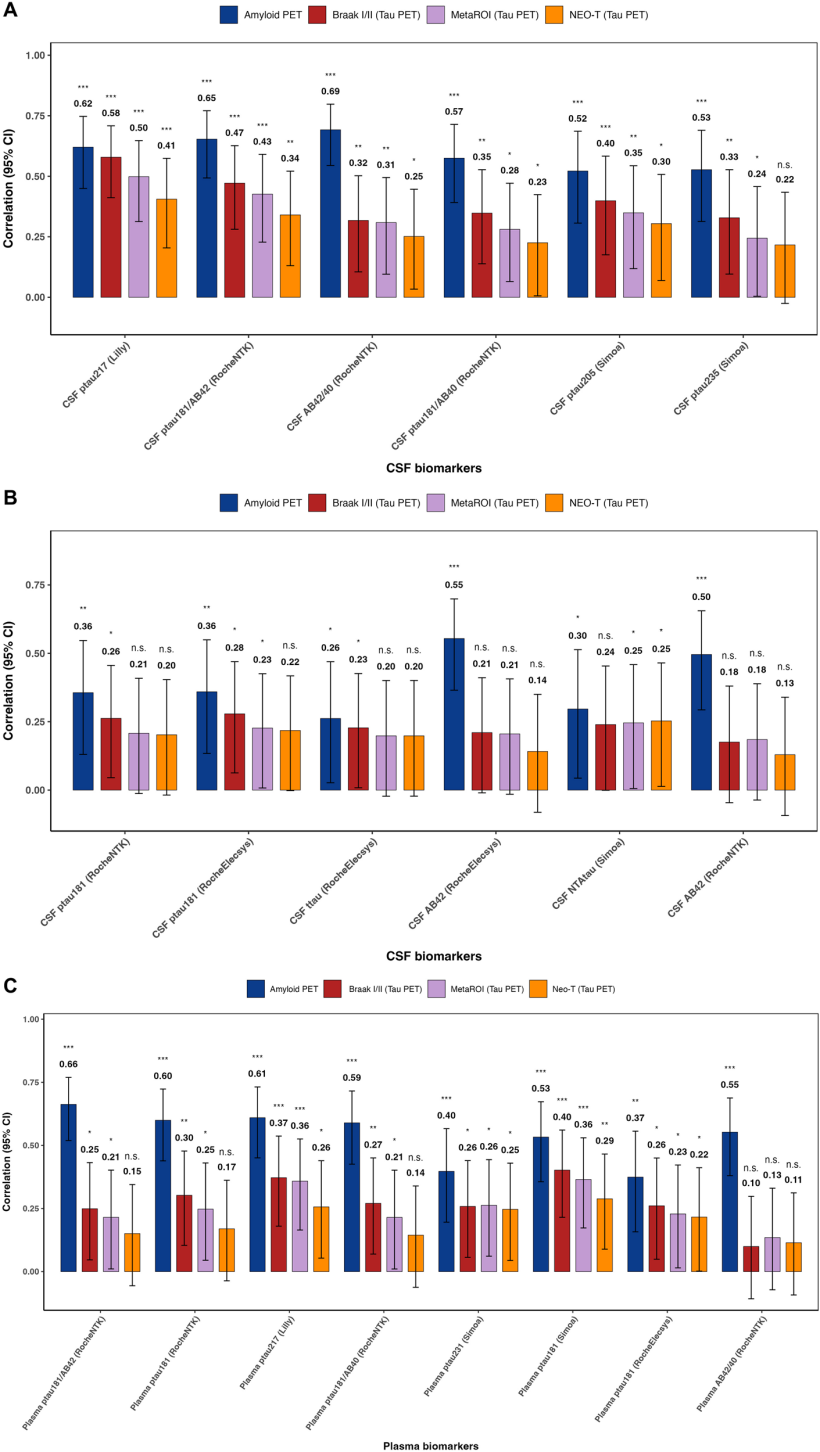
Partial correlations (with 95% confidence intervals and *p* values) between amyloid burden (Centiloid values) (navy bar), tau PET standardized uptake value ratio in Braak I/II region (red bar), temporal MetaROI (purple bar), and NEO‐T (orange bar) and (A and B) cerebrospinal fluid biomarkers and (C) plasma biomarkers. All analyses were adjusted for age, sex, *APOE ε4* status, and the time interval between PET imaging and fluid biomarker collection. To harmonize directionality, correlations of tau positron emission tomography with amyloid beta (Aβ) 42/40 and Aβ42 were sign‐flipped (multiplied by −1) for display only.

Figure 2 shows the partial correlations between CL, tau PET Braak I/II, temporal MetaROI, and NEO‐T with CSF‐ and plasma‐based AD biomarkers, adjusting for age, sex, *APOE ε4* status, and the time interval between PET imaging and fluid biomarker collection. For the correlations between tau PET outcomes (Braak I/II, MetaROI, and NEO‐T), the time interval was statistically significant, likely due to the longer time interval between measurements. In contrast, for CL, the time interval was not statistically significant, as the plasma biomarkers were measured more concurrently with amyloid PET. CSF p‐tau217 showed the strongest association with amyloid burden (CL: *r* = 0.62, 95% CI: 0.45 to 0.75, *p* < 0.001) and moderate associations with tau PET measures (Braak I/II: *r* = 0.58, 95% CI: 0.41 to 0.71, *p* < 0.001; temporal MetaROI: *r* = 0.50, 95% CI: 0.31 to 0.65, *p* < 0.001). CSF p‐tau181/Aβ42 was similarly robust for amyloid (*r* = 0.65, 95% CI: 0.49 to 0.77, *p* < 0.001) and showed moderate correlations with Braak I/II (*r* = 0.47, 95% CI: 0.28 to 0.63, *p* < 0.001) and MetaROI (*r* = 0.43, 95% CI: 0.23 to 0.59, *p* < 0.001). It should be noted that p‐tau181/Aβ42 and p‐tau181/Aβ40 consistently show stronger associations with CL and tau PET than p‐tau181 alone, indicating that combining tau with Aβ enhances association strength.

Among plasma biomarkers, the strongest association with amyloid PET was for p‐tau181/Aβ42 (*r* = 0.66, 95% CI: 0.52 to 0.77, *p* < 0.001), followed closely by p‐tau217 (*r* = 0.61, 95% CI: 0.45 to 0.73, *p* < 0.001). For tau PET, the largest effects were seen for Braak I/II with plasma p‐tau217 (*r* = 0.37, 95% CI: 0.18 to 0.54, *p* < 0.001). For the temporal MetaROI, p‐tau217 again performed best (*r* = 0.36, 95% CI: 0.16 to 0.53, *p* < 0.001). Correlations between NEO‐T and fluid biomarkers were substantially weaker than those observed for Braak I/II and the temporal MetaROI. Additionally, plasma p‐tau181 (*r* = 0.34, 95% CI: 0.07 to 0.60, *p* < 0.001), p‐tau217 (*r* = 0.29, 95% CI: 0.04 to 0.55, *p* < 0.001), and p‐tau231 (*r *= 0.27, 95% CI: 0.04 to 0.53, *p* < 0.001) each showed moderate correlation with Braak III/IV tau PET SUVR. Among the CSF biomarkers, p‐tau217 showed the strongest correlation with Braak III/IV SUVR (*r* = 0.46, 95% CI: 0.08 to 0.72, *p* < 0.001), followed by p‐tau181/Aβ42 (*r* = 0.38, 95% CI: 0.09 to 0.62, *p* < 0.001) (Table S2). Notably, among the three p‐tau181 platforms, only Simoa plasma p‐tau181 showed correlations comparable to or exceeding p‐tau217 with tau‐PET outcomes, including Braak I/II, temporal MetaROI, and Braak III/IV, whereas RocheNTK and RocheElecsys plasma p‐tau181 displayed weaker or non‐significant associations. None of the fluid biomarkers showed significant correlations with either Braak V/VI or global tau PET SUVR. Similarly, no statistically significant correlations were found between tau PET outcomes and cognitive measures.

### 3.3 ROC analysis

Tables S3 and 2 present the performance of fluid biomarkers in predicting Braak I/II tau PET positivity, unadjusted and adjusted for age, sex, and *APOE ɛ4* carriership, respectively. Comparison of the AUCs between unadjusted and adjusted models indicates that biomarker performance (AUC and 95% CI) improves with adjustment; however, the overall pattern remains consistent for most biomarkers. Notably, the plasma Aβ42/40 ratio and CSF p‐tau235 do not demonstrate statistically significant performance in the unadjusted models. Table 2 summarizes the performance of fluid biomarkers and amyloid PET for predicting Braak I/II tau PET positivity, along with their respective positivity thresholds. Additionally, each biomarker's PPV and NPV are reported. Models incorporating age, sex, and *APOE ɛ4* alone had an AUC of 0.78 (95% CI: 0.61 to 0.95, *p* < 0.005), serving as the baseline reference for biomarker evaluation. Adding fluid biomarkers/CL to the model improved the performance and resulted in higher AUC for the biomarkers (Figure S6). In brief, among plasma biomarkers, p‐tau181 demonstrated the highest (AUC = 0.86, 95% CI: 0.72 to 1.00, *p* < 0.0001). Other plasma biomarkers, including p‐tau217 (AUC = 0.84, 95% CI: 0.68 to 0.99, *p* < 0.001), and p‐tau181/Aβ42 (AUC = 0.84, 95% CI: 0.65 to 0.99, *p* < 0.001), also showed strong predictive performance. Among CSF biomarkers, p‐tau181/Aβ42 and p‐tau217 exhibited the highest AUC of 0.94 (95% CI: 0.85 to 1.00, *p* < 0.0001) and 0.93 (0.95% CI: 0.85 to 1.00, *p* < 0.0001), respectively.

**TABLE 2:** AUC (95% CI), corresponding *p* value, YI, positivity threshold, PPV, and NPV of core AD fluid biomarkers and Centiloid for predicting Braak I/II positivity, extracted from ROC analysis.

Biomarker	AUC	95% CI	*P* value	YI	Positivity threshold	PPV	NPV
*Age + sex + APOE ɛ4*	0.78	0.61 to 0.95	<0.005	–	–	–	–
*Plasma A*β*42/40 (RocheNTK) + Age + Sex + APOE ɛ4*	0.80	0.63 to 0.96	<0.005	0.48	0.12	0.14	0.98
*Plasma p‐tau231 (Simoa) + Age + Sex + APOE ɛ4*	0.82	0.65 to 0.99	<0.001	0.51	14.21	0.21	0.95
*Plasma p‐tau181 (RocheNTK) + Age + Sex + APOE ɛ4*	0.84	0.68 to 0.99	<0.001	0.53	0.89	0.25	0.96
*Plasma p‐tau181/A*β*42 (RocheNTK) + Age + Sex + APOE ɛ4*	0.84	0.69 to 0.99	<0.001	0.54	0.027	0.20	0.95
*Plasma p‐tau217 (Lilly) + Age + Sex + APOE ɛ4*	0.84	0.68 to 0.99	<0.001	0.47	0.20	0.24	0.96
*Plasma p‐tau181/A*β*40 (RocheNTK) + Age + Sex + APOE ɛ4*	0.85	0.70 to 0.99	<0.001	0.54	0.003	0.21	0.96
*Plasma p‐tau181 (Simoa) + Age + Sex + APOE ɛ4*	0.86	0.72 to 1.00	<0.0001	0.62	26	0.25	0.96
*CSF p‐tau235 (Simoa) + Age + Sex + APOE ɛ4*	0.89	0.71 to 1.00	<0.005	0.56	21.33	0.18	0.97
*CSF NTAtau + Age + Sex + APOE ɛ4*	0.90	0.76 to 1.00	<0.001	0.60	60.09	0.16	0.98
*Centiloid + Age + Sex + APOE ɛ4*	0.91	0.82 to 1.00	<0.0001	0.67	38.17	0.33	0.99
*CSF A*β*42/40 ratio + Age + Sex + APOE ɛ4*	0.91	0.81 to 1.00	<0.0001	0.64	0.085	0.09	1.00
*CSF p‐tau181/A*β*40 + Age + Sex + APOE ɛ4*	0.91	0.81 to 1.00	<0.0001	0.65	0.001	0.14	1.00
*CSF p‐tau205 (Simoa) + Age + Sex + APOE ɛ4*	0.93	0.83 to 1.00	<0.0001	0.68	2.45	0.21	1.00
*CSF p‐tau217 (Lilly) + Age + Sex + APOE ɛ4*	0.93	0.84 to 1.00	<0.0001	0.66	11.83	0.24	1.00
*CSF p‐tau181/A*β*42 + Age + Sex + APOE ɛ4*	0.94	0.85 to 1.00	<0.0001	0.62	0.029	0.20	0.98

Abbreviations: Aβ, amyloid beta; AD, Alzheimer's disease; *APOE*, apolipoprotein E; AUC, area under the curve; CSF, cerebrospinal fluid; NPV, negative predictive value; PPV, positive predictive value; p‐tau, phosphorylated tau; ROC, receiver operating characteristic; YI, Youden Index.

PPV values for all biomarkers ranged from low to moderate, with CL demonstrating the highest PPV (0.33), indicating slightly better predictive value for detecting Braak I/II positivity compared to fluid biomarkers, such as CSF and plasma p‐tau217 (PPV = 0.24). Using our tau PET staging, 90.9% of participants were classified as negative, and applying the 0.2 cut‐off for plasma p‐tau217 similarly identified 96% as negative. This high proportion of tau PET negative individuals likely accounts for the strong NPVs observed across all biomarkers, each exceeding 0.94.

### 3.4 PET‐based staging

To establish PET‐based staging, we applied positivity thresholds derived from ROC analyses (Table 2). Amyloid PET positivity (A status) was defined using a CL cut‐off of 38.17, while tau PET positivity (T status) was determined using a Braak I/II SUVR threshold of 1.36. Participants with CL values between 12 and 38.17 were classified as being in the amyloid PET gray zone (Gz). Based on these PET‐derived thresholds, participants were grouped as follows:

PET (A‐T‐): Centiloid < 12 and Braak I/II < 1.35
PET (A(Gz)T‐): 12≤Centiloid < 38.17 and Braak I/II < 1.35
PET (A+T‐): Centiloid≥38.17 and Braak I/II < 1.35
PET(A+T+): Centiloid≥38.17 and Braak I/II≥1.35

It is worth noting that, under the PET‐based staging criteria, no participants were classified as PET (A−T+) or PET (A(Gz)T+). The demographic characteristics of the participants in each PET‐based AT staging group are shown in Table S4.

**FIGURE 3:**
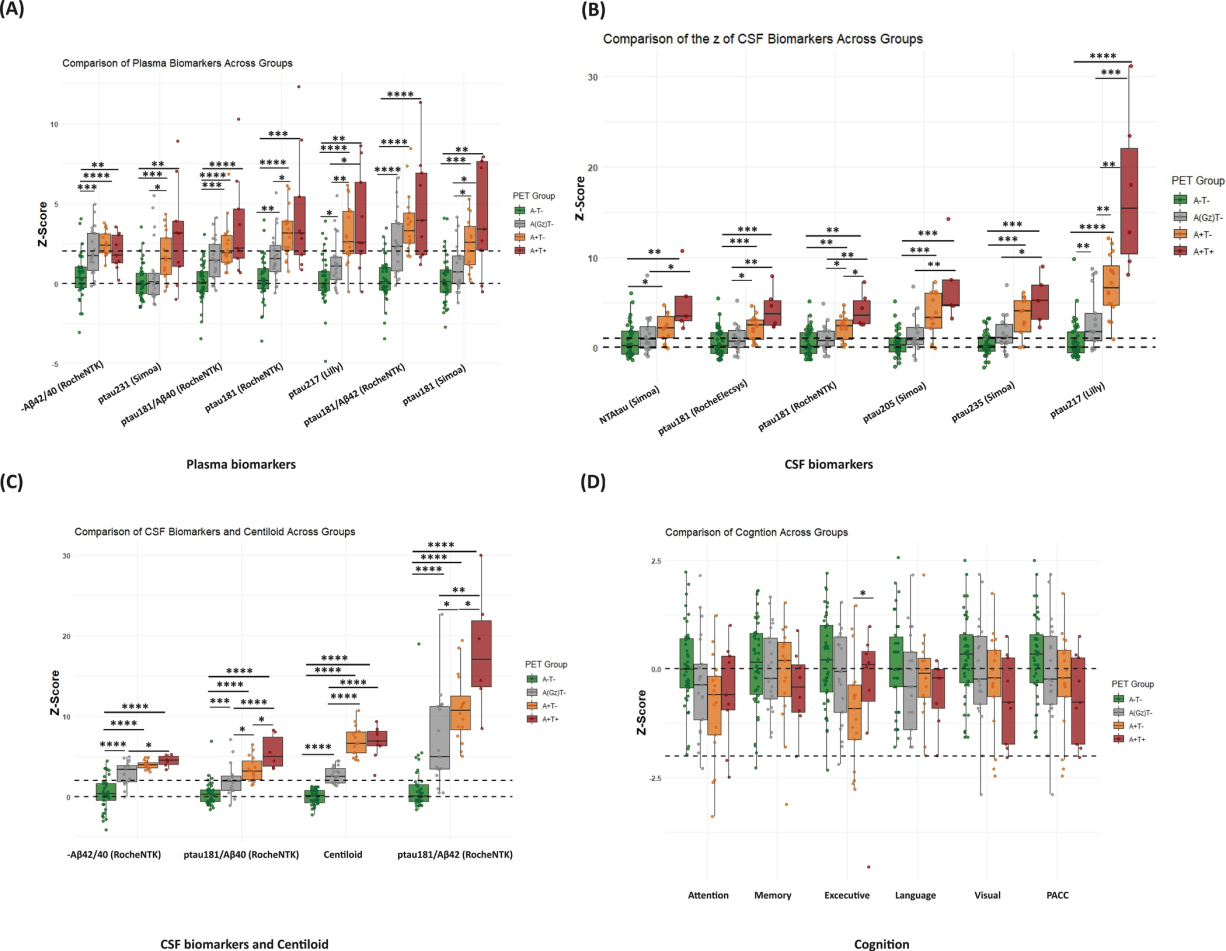
Group‐wise comparisons of plasma, cerebrospinal fluid cerebrospinal fluid (CSF), imaging biomarkers, and cognitive composites across positron emission tomography (PET)‐defined groups. (A) Plasma biomarker *z*‐scores across PET(A−T−), PET(AGZT−), PET(A+T−), and PET(A+T+). (B) CSF phosphorylated tau (p‐tau) biomarker *z*‐scores across the same groups. (C) Additional CSF and Alzheimer's disease (AD) imaging biomarker *z*‐scores across PET‐defined groups. (D) *Z*‐scored cognitive composite measures across the PET‐defined groups. Statistical comparisons between groups are indicated, with significant differences denoted by *p* values. ** Note that the *z*‐scores of the Aβ42/40 ratios were multiplied by −1 for clearer presentation.

**FIGURE 4:**
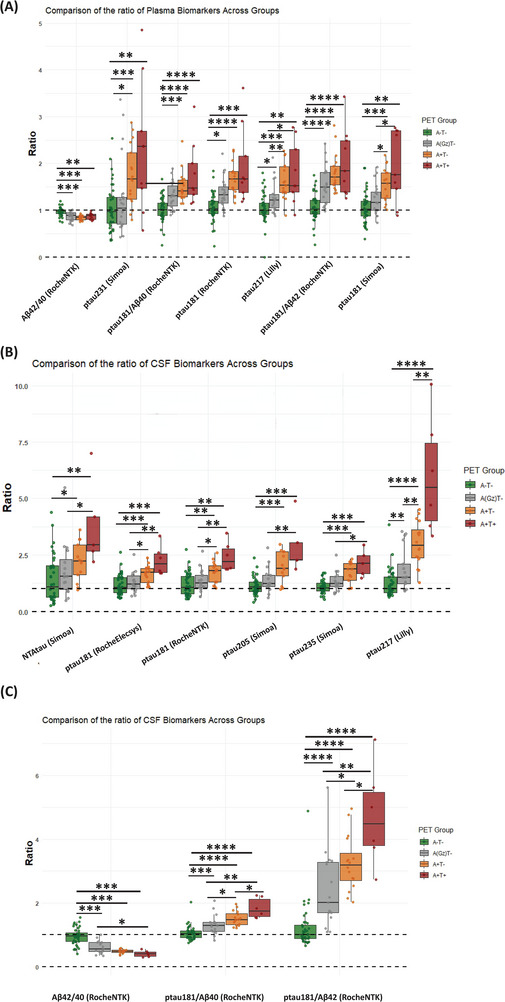
Group‐wise comparisons of biomarker ratios relative to positron emission tomography (PET)‐based reference group. The figure illustrates the fold changes in plasma and CSF biomarkers across PET‐defined stages, including PET(A−T−), PET(A[GZ]T−), PET(A+T−), and PET(A+T+), respectively, highlighting significant between‐group differences.

Figure 3 shows the comparison between fluid biomarkers, CL, and cognition as a function of the PET‐derived staging. Plasma biomarker levels demonstrated a stepwise increase across PET‐defined groups, with the highest values observed in the A+T+ group. Significant differences between PET‐defined stages were observed for both plasma p‐tau181 assays and plasma p‐tau217, demonstrating the pronounced stage‐dependent elevations. Notably, plasma p‐tau181/Aβ42 also showed significant elevations in A+T+ individuals, reflecting their strong association with amyloid and tau pathology. CSF p‐tau217 and p‐tau181/Aβ42 presented the highest dynamic range and differentiation across different PET‐based stages, followed by p‐tau235 and p‐tau205. Although cognitive scores showed a stepwise decline with progression to more advanced stages, the between‐group differences did not reach statistical significance after applying Bonferroni correction for multiple comparisons (Figure 3D). When comparing the fold changes across different plasma biomarkers, p‐tau181, p‐tau181/Aβ42, p‐tau217, p‐tau231 showed an approximately two‐fold increase in the PET(A+T+) group relative to the reference (A−T−) group (Figure 4A). Most CSF biomarkers showed increased fold changes across PET stages, with p‐tau217 and p‐tau181/Aβ42 having the highest increases, about 4.5‐ and 5.5‐fold in the PET(A+T+) group. NTAtau showed an ∼threefold rise in the PET(A+T+) group compared to PET(A−T−) (Figure 4B,C). The statistical report for between‐group comparison for both *z*‐scored biomarkers and ratio can be found in Tables S5–S9.

## 4 DISCUSSION

In this study, we used tau PET to investigate NFT pathology in the earliest preclinical stages of the AD continuum and the associations with amyloid Centiloids, fluid‐based AD biomarkers in plasma (Aβ42/40, p‐tau181, p‐tau181/Aβ42, p‐tau181/Aβ40, p‐tau217, and p‐tau231) and CSF (Aβ42/40, p‐tau181, p‐tau181/Aβ42, p‐tau181/Aβ40, p‐tau205, p‐tau235, and NTAtau). To comprehensively characterize these associations, we also examined relationships with amyloid pathology using amyloid PET and cognitive performance.

Our findings reveal that tau PET signal is elevated during preclinical AD, with tau positivity in Braak I/II emerging in less than half of the individuals with CL above 38.^17^, ^18^ This suggests that NFT pathology can emerge earlier than previously recognized in the AD continuum, as prior reports have typically described tau positivity at CL values exceeding 50 CL.^46^, ^47^ Moreover, staging of the ^18^F‐RO‐948 PET scans in our sample aligned with the Braak hierarchical staging model for most participants with tau positivity. Previous work showed heterogeneity beyond Braak staging in the tau PET spread.^13^, ^14^ However, in our work, the “limbic” subtype, which mostly corresponds to the pattern of tau spread in the Braak model, was associated with *APOE ε4* carriership. The high prevalence of *APOE ε4* carriers in our sample may explain the strong conformance to the Braak model observed in our cohort. However, observing two participants with the medial temporal lobe (MTL)‐sparing tau pattern reveals that distinct tau propagation patterns can already be detected even in the early stages. Beyond Braak staging, tau PET positivity was assessed using temporal MetaROI, NEO‐T (per NIA‐AA recommendations),^48^, ^49^ and CenTauR‐z methods. Positivity in temporal MetaROI and NEO‐T largely overlapped with Braak III/IV, indicating consistency across composites. In contrast, the CenTauR‐z method assigned a z‐score ≥ 2 to some tau PET scans from participants classified as A−T− and A+T−, despite their CL values being low to moderate (CL < 30). Discrepancies likely reflect methodological differences, especially ROI definitions. Consistent with the CenTauR‐z study, which robustly separates low from high tau, we find that in early accumulation subject‐specific Braak ROIs better capture subtle tau changes.^33^ Moreover, our tau PET pipeline further enhances early detection by modeling meningeal uptake and minimizing off‐target spillover into cortical ROIs.

Plasma p‐tau biomarkers demonstrated a moderate association with Braak I/II and temporal MetaROI SUVRs, with p‐tau217 showing the strongest correlations, even after adjusting for age, sex, and *APOE ε4* carriership. For amyloid PET CL measures, plasma p‐tau181/Aβ42 exhibited the strongest association, followed by p‐tau217 and p‐tau181/Aβ40, with comparable performance. As expected, CSF biomarkers had stronger correlations with both amyloid and tau PET signals, with CSF p‐tau217 and p‐tau181/Aβ42 showing the highest associations, followed by p‐tau181/Aβ40. Additionally, both plasma and CSF p‐tau217 showed moderate associations with Braak III/IV SUVR.

In our analyses, correlations between fluid biomarkers and amyloid PET were generally stronger than with tau PET. *Post hoc* comparisons showed no significant differences across CSF p‐tau epitope correlations with CL, Braak I/II, and MetaROI.^50^ Nonetheless, CSF p‐tau205 and p‐tau235 showed modest associations with early Braak regions, suggesting these epitopes begin to change during initial phases of tau accumulation. Notably, while prior work associated p‐tau205 predominantly with advanced Braak V/VI stages, we observed moderate correlations with earlier stages including Braak I/II and MetaROI.^51^, ^52^ By contrast, for plasma p‐tau biomarkers, correlations with tau‐PET measures were significantly weaker than those with amyloid PET.^20^, ^53^ A plausible explanation is that our cohort consists of cognitively unimpaired individuals at the preclinical stage of AD, where Aβ‐related changes are more prevalent (48/99 with CL ≥ 12; 48.5%), with a higher dynamic range than overt tau aggregation (Braak I/II‐positive:9/99, 9.1%; MetaROI‐positive:7/99, 7.1%), limiting dynamic range and attenuating correlations with tau PET.

For fluid biomarkers and amyloid PET CL, our ROC analysis showed good to excellent AUCs when entering age, sex, and *APOE ε4* status in the model. Despite the high NPV of all the biomarkers (>0.94), their PPV remained low, with CL achieving the highest PPV (0.33), followed by plasma and CSF p‐tau biomarkers (∼0.24 to 0.25). These low PPVs reflect the low prevalence of tau PET positivity in our cohort: low prevalence increases NPV and reduces PPV at a given threshold. Accordingly, at the operational cut‑point used here, only about 25% of participants testing positive on the best‑performing plasma biomarkers are expected to be tau PET positive at these early AD stages. Conversely, when combined with demographic and genetic information, the models yield very high NPV (>95%) for ruling out tau PET positivity, which supports their use for screening. Note that while low prevalence inflates NPV, ROC AUC is, in principle, prevalence‑independent.

Participants were classified into four amyloid/tau PET groups, including a “gray zone,” to test how fluid biomarkers track early fibrillar AD pathology. The gray zone was set to 12 to 38 CL, where the lower bound comes from prior studies^5^, ^45^, ^54^ and the upper bound was derived by our ROC analysis, aligning with a previously reported threshold for the onset of neocortical tau proliferation.^55^ Including this group highlights dynamic early trajectories and their effects on plasma/CSF biomarkers, which is particularly relevant given the higher gray‐zone prevalence in preclinical cohorts. Importantly, all participants adhered to this classification, with none being tau PET‐positive while amyloid‐negative or in the amyloid gray zone, ensuring alignment with the AD continuum. Our positivity threshold was defined as mean plus two standard deviations (mean + 2 SD) of regional Braak ROI uptake in a reference group of cognitively unimpaired, middle‐aged individuals who were A−T− based on CSF biomarkers. The rationale for selecting reference group based on CSF AT status lies in the expectation that CSF biomarkers tend to become abnormal earlier than PET, yielding a more sensitive definition.^56^ The consistency of this method is further supported by the resulting staging: No individuals classified as CSF A−T− were tau PET positive, and all tau PET‐positive individuals had CL values above 38. Importantly, the design of our staging method did not exclude amyloid PET negative, tau PET‐positive cases by definition, as the thresholds for abnormality were determined independently for CSF‐based amyloid and tau biomarkers. Our findings highlight that CSF p‐tau217 and p‐tau181/Aβ42 exhibited the greatest dynamic range and the largest fold increases across PET‐based AD stages, with a clear stepwise rise as pathology progressed. Among plasma biomarkers, p‐tau181, p‐tau181/Aβ42, and p‐tau217 also showed step‐wise increases across stages, though with lower fold changes compared to their CSF counterparts.

Furthermore, our analysis of biomarker fold changes across PET‐based stages demonstrated that CSF p‐tau217 and p‐tau181/Aβ42 had the most pronounced increases, particularly in the PET(A+T+) stage, reinforcing their strong association with tau pathology and amyloid plaques. Although plasma p‐tau217 showed weaker performance than its CSF counterpart, it still exhibited a ∼1.5‐fold increase in the more advanced stages of pathology compared to PET(A−T−), confirming its alteration due to the core‐AD pathology.

These results were obtained in a sample with a relatively balanced distribution of participants in each CSF‐based AT stage, as determined by study design. It is essential to highlight that participants in this study are in the early stage of AD, all classified as CU individuals. Their mean age (65.15 ± 5.00), as well as their amyloid load measured by PET (CL = 22.52 ± 26.25), is considerably lower than in other studies found in the literature that include CU individuals. These results support that the sample studied is at relatively early stages of the preclinical AD continuum and can also explain the lack of associations observed between tau PET and cognitive performance. This sample, being at the earliest preclinical AD continuum, can be considered a strength of the study. In addition, participants have been thoroughly characterized with several fluid and imaging biomarkers, showcasing the interrelationships of these biomarkers in the earliest pathological AD continuum.

On the other hand, this study is not free of limitations. The sample was highly selected and enriched for AD risk factors, particularly amyloid, as reflected in the high percentage of *APOE ε4* carriers. While this may influence tau prevalence estimates and the detection of certain tau subtypes compared to unselected populations, it is unlikely to significantly affect the associations between tau PET and other AD biomarkers, which should remain broadly generalizable. However, the small number of tau‐positive cases limits the dynamic range and statistical power. Effect sizes should therefore be interpreted cautiously. Moreover, the cross‐sectional nature of the analyses does not allow us to study several interesting questions, such as the progression of tau spread over time or the capacity of fluid biomarkers to predict tau accumulation in the future. In this regard, the longitudinal collection of ^18^F‐RO‐948 in this sample is under way and will allow us to address these questions in the future.

To summarize, ^18^F‐RO‐948 PET could detect NFT pathology in participants of the ALFA+ cohort who are cognitively unimpaired and at the earliest preclinical AD stages. Most of the positive cases conformed to the Braak hierarchical model. Despite the association between the tau PET SUVRs and fluid biomarkers, the fluid biomarkers studied here, and more specifically plasma biomarkers, show limited capacity to detect tau PET positivity. On the other hand, plasma biomarkers display a very high capacity to rule out tau PET positivity when combined with demographics and *APOE4 ε4* status, which may bring value for screening purposes.

## CONFLICT OF INTEREST STATEMENT

Theresa A. Day is a full‐time employee and stockholder of Eli Lilly and Company, Indianapolis, IN, USA. Jeffrey L Dage (JLD) is an inventor on patents or patent applications assigned to Eli Lilly and Company relating to the assays, methods, reagents, and/or compositions of matter for p‐tau assays and Aβ‐targeting therapeutics. JLD has/is served/serving as a consultant or on advisory boards for Eisai, Abbvie, Genotix Biotechnologies Inc., Gates Ventures, Gate Neurosciences, Dolby Family Ventures, Karuna Therapeutics, Alzheimer's Drug Discovery Foundation (ADDF), ALZpath Inc., Cognito Therapeutics, Inc., Eli Lilly and Company, Prevail Therapeutics, Neurogen Biomarking, Spear Bio, Rush University, University of Kentucky, Tymora Analytical Operations, and Quanterix. JLD has received research support from ADx Neurosciences, Fujirebio, Roche Diagnostics, and Eli Lilly and Company in the past 2 years. JLD has received speaker fees from Eli Lilly and Company and LabCorp. JLD is a founder and advisor for Monument Biosciences and Dage Scientific LLC. JLD has stock or stock options in Eli Lilly and Company, Genotix Biotechnologies, AlzPath Inc., Neurogen Biomarking, and Monument Biosciences. Thomas Karikari (TKK) has consulted for Quanterix Corporation, SpearBio Inc., Neurogen Biomarking LLC, and Alzheon, and has served on advisory boards for Siemens Healthineers, Neurogen Biomarking LLC, and Alzheon (which may come with minority stock equity interest/stock options), outside the submitted work. He has received in‐kind research support from Janssen Research Laboratories, SpearBio Inc., and Alamar Biosciences, as well as meeting travel support from the Alzheimer's Association and Neurogen Biomarking LLC, outside the submitted work. TKK has received royalties from Bioventix for the transfer of specific tau antibodies and assays to third‐party organizations. He has received honoraria for speaker/grant review engagements from the NIH, UPENN, UW‐Madison, the Cherry Blossom symposium, the HABS‐HD/ADNI4 Health Enhancement Scientific Program, Advent Health Translational Research Institute, Brain Health conference, Barcelona‐Pittsburgh conference, the International Neuropsychological Society, the Icahn School of Medicine at Mount Sinai, and the Quebec Center for Drug Discovery, Canada, all outside of the submitted work. TKK serves/has served as a guest editor for npj Dementia, as an invited member of the World Health Organization committee to develop preferred product characteristics for blood‐based biomarker diagnostics for AD, as an executive committee member for the Human Amyloid Imaging (HAI) conference, as an elected member of the NACC ADRCs Steering Committee, as co‐director of the NACC ADRCs Biofluid Biomarker Working Group, and as a member of the Alzheimer's Association committees to develop Appropriate Use Criteria for clinical use of blood‐based biomarkers, and treatment‐related amyloid clearance. TKK is an inventor on several patents and provisional patents regarding biofluid biomarker methods, targets, and reagents/compositions that may generate income for the institution and/or self should they be licensed and/or transferred to another organization. These include WO2020193500A1: Use of a ps396 assay to diagnose tauopathies; 63/679,361: Methods to Evaluate Early‐Stage Pre‐Tangle TAU Aggregates and Treatment of Alzheimer's Disease Patients; 63/672,952: Method for the Quantification of Plasma Amyloid‐Beta Biomarkers in Alzheimer's Disease; 63/693,956: Anti‐tau Protein Antigen Binding Reagents; and 2450702‐2: Detection of oligomeric tau and soluble tau aggregates. Matteo Tonietto, Edilio Borroni, and Gregory Klein are full‐time employees of F. Hoffmann‐La Roche, Basel, Switzerland. Margherita Carboni and Clara Quijano‐Rubio are full‐time employees of Roche Diagnostics International, Rotkreuz, Switzerland. Gwendlyn Kollmorgen is a full‐time Roche Diagnostics GmbH employee. Eugeen Vanmechelen is a full‐time employee of ADx NeuroSciences, Ghent, Belgium. José Luis Molinuevo is a full‐time employee of Novartis, Basel, Switzerland. Henrik Zetterberg (H.Z.) is a Wallenberg Scholar and a Distinguished Professor at the Swedish Research Council supported by grants from the Swedish Research Council (#2023‐00356, #2022‐01018 and #2019‐02397), the European Union's Horizon Europe research and innovation programme under grant agreement No 101053962, Swedish State Support for Clinical Research (#ALFGBG‐71320), the ADDF, USA (#201809‐2016862), the AD Strategic Fund and the Alzheimer's Association (#ADSF‐21‐831376‐C, #ADSF‐21‐831381‐C, #ADSF‐21‐831377‐C, and #ADSF‐24‐1284328‐C), the European Partnership on Metrology, co‐financed by the European Union's Horizon Europe Research and Innovation Programme, and by the Participating States (NEuroBioStand, #22HLT07), the Bluefield Project, Cure Alzheimer's Fund, the Olav Thon Foundation, the Erling‐Persson Family Foundation, Familjen Rönströms Stiftelse, Stiftelsen för Gamla Tjänarinnor, Hjärnfonden, Sweden (#FO2022‐0270), the European Union's Horizon 2020 research and innovation programme under the Marie Skłodowska‐Curie grant agreement 860197 (MIRIADE), the European Union Joint Programme – Neurodegenerative Disease Research (JPND2021‐00694), the National Institute for Health and Care Research University College London Hospitals Biomedical Research Centre, the UK Dementia Research Institute at UCL (UKDRI‐1003), and an anonymous donor. H.Z. has served on scientific advisory boards and/or as a consultant for Abbvie, Acumen, Alector, Alzinova, ALZpath, Amylyx, Annexon, Apellis, Artery Therapeutics, AZTherapies, Cognito Therapeutics, CogRx, Denali, Eisai, Enigma, LabCorp, Merry Life, Nervgen, Novo Nordisk, Optoceutics, Passage Bio, Pinteon Therapeutics, Prothena, Quanterix, Red Abbey Labs, reMYND, Roche, Samumed, Siemens Healthineers, Triplet Therapeutics, and Wave, has given lectures sponsored by Alzecure, BioArctic, Biogen, Cellectricon, Fujirebio, Lilly, Novo Nordisk, Roche, and WebMD, and is a co‐founder of Brain Biomarker Solutions in Gothenburg AB (BBS), which is a part of the GU Ventures Incubator Program (outside submitted work). Kaj Blennow is supported by the Swedish Research Council (#2017‐00915 and #2022‐00732), the Swedish Alzheimer Foundation (#AF‐930351, #AF‐939721 and #AF‐968270), Hjärnfonden, Sweden (#FO2017‐0243 and #ALZ2022‐0006), the Swedish state under the agreement between the Swedish government and the County Councils, the ALF‐agreement (#ALFGBG‐715986 and #ALFGBG‐965240), the Alzheimer's Association 2021 Zenith Award (ZEN‐21‐848495), and the Alzheimer's Association 2022‐2025 Grant (SG‐23‐1038904 QC). Marc Suárez‐Calvet receives funding from the European Research Council (ERC) under the European Union's Horizon 2020 research and innovation programme (Grant agreement No. 948677), Project “PI19/00155,” funded by Instituto de Salud Carlos III (ISCIII) and co‐funded by the European Union, and from a fellowship from “la Caixa” Foundation (ID 100010434) and from the European Union's Horizon 2020 research and innovation programme under the Marie Skłodowska‐Curie grant agreement No 847648 (LCF/BQ/PR21/11840004). Thomas K. Karikari was supported by grants 1 R01 AG083874‐01 and 1U24AG082930 from the National Institutes of Health (NIH), the Swedish Research Council (Vetenskåpradet; 2021‐03244), the Alzheimer's Association (AARF‐21‐850325), the Swedish Alzheimer Foundation (Alzheimerfonden), the Aina (Ann) Wallströms and Mary‐Ann Sjöbloms Stiftelsen, and the Emil och Wera Cornells stiftelsen. Juan Domingo Gispert (JDG) was supported by the Spanish Ministry of Science and Innovation (RYC‑2013‑13054). JDG has also received research support from the EU/EFPIA Innovative Medicines Initiative Joint Undertaking AMYPAD (grant agreement 115952), EIT Digital (grant 2021), and Ministerio de Ciencia y Universidades (grant agreement RTI2018‑102261). JDG is currently full‐time employee at AstraZeneca, and has received research support from GE Healthcare, Roche Diagnostics, Hoffmann ‐ La Roche, Life‐Molecular Imaging and Philips Netherlands, speaker/consulting fees from Biogen, Roche Diagnostics, Life‐Molecular Imaging and Esteve, participated in the Molecular Neuroimaging Scientific Advisory Board of Prothena Biosciences, and is the founder and co‐owner of BetaScreen SL. Gonzalo Sánchez‐Benavides receives funding from the Ministerio de Ciencia e Innovación, Spanish Research Agency, PID2020‐119556RA‐I00; and the grant CP23/00039, funded by the Instituto de Salud Carlos III (ISCIII) and co‐funded by the European Union/FSE+. Oriol Grau‐Rivera receives funding from the Alzheimer's Association Research Fellowship Program (2019‐AARF‐644568), from Instituto de Salud Carlos III (PI19/00117); the Spanish Ministry of Science, Innovation and Universities (Juan de la Cierva programme IJC2020‐043417‐I); and the grant IJC2020‐043417‐I, funded by MCIN/AEI/10.13039/501100011033 and the European Union NextGenerationEU/PRTR. Anna Brugulat‐Serrat receives funding from the Alzheimer's Association through the Alzheimer's Association Clinician Scientist Fellowship (AACSF‐23‐1145154). Author disclosures are available in the Supporting Information.

## DISCLAIMER

This article reflects the views of the authors, and the associations are not liable for any use that may be made of the information contained herein.

## CONSENT

The ALFA study was conducted in accordance with the directives of the Spanish Law 14/2007, of July 3, on Biomedical Research (Ley 14/2007 de Investigación Biomédica). The ALFA study protocol was approved by the Independent Ethics Committee Parc de Salut Mar, Barcelona, and registered at ClinicalTrials.gov (Identifier: NCT01835717). All participants accepted the study procedures by signing the study's informed consent form, which had also been approved by the same Institutional Review Board.

## Supporting information



Supporting Information

Supporting Information

## Data Availability

All requests for raw and analyzed data and materials will be promptly reviewed by the corresponding authors and the Barcelonaβeta Brain Research Center to verify whether the request is subject to any intellectual property or confidentiality obligations. Bulk anonymized data can be shared by request from any qualified investigator for the sole purpose of replicating procedures and results presented in the article, provided that data transfer is in agreement with EU legislation.
