# Supplementary material for: Associations of 18F‐RO‐948 tau PET with fluid AD biomarkers, Centiloid, and cognition in early AD continuum
Source: Alzheimers Dement. 2026 Apr 17;22(4):e71176. doi: 10.1002/alz.71176 (PMC13088146; doi:10.1002/alz.71176)
Supplement: Supplementary file 1 — Supporting Information [file ALZ-22-e71176-s002.pdf]

Associations of ^{18}F-RO-948 Tau PET with Fluid AD Biomarkers, Centiloid, and Cognition in the Early AD Continuum

Mahnaz Shekari[1,2,3], Armand González Escalante[1,2,3], Marta Milà-Alomà[1,2,4], Carles Falcon[1,2,5], David López-Martos[1,2], Gonzalo Sánchez-Benavides[1,2,4], Anna Brugulat-Serrat[1,2,4,6], Aida Niñerola-Baizán[5,7], Nicholas J. Ashton[9-11], Thomas K. Karikari[9,12], Juan Lantero-Rodriguez[9], Laia Montoliu-Gaya[9], Anniina Snellman[9,13], Theresa A Day[14], Jeffrey L. Dage[15], Paula Ortiz-Romero[1,2], Matteo Tonietto[8], Edilio Borroni[8], Gregory Klein[8], Gwendlyn Kollmorgen[16], Margherita Carboni[17], Clara Quijano-Rubio[17], Eugeen Vanmechelen[18], Carolina Minguillón[1,2,4], Karine Fauria[1,4], Andrés Perissinotti[5,7], José Luis Molinuevo[1,19], Henrik Zetterberg[20-23], Kaj Blennow[20,21], Oriol Grau-Rivera[1,2,4,24], Marc Suárez-Calvet[1,2,4,24], Juan Domingo Gispert[1,25], for the ALFA Study.

[1]Barcelonaβeta Brain Research Center (BBRC), Pasqual Maragall Foundation. Barcelona, Spain.
[2]Hospital del Mar Research Institute (HMRI), Barcelona, Spain.
[3]Universitat Pompeu Fabra, Barcelona, Spain.
[4]Centro de Investigación Biomédica en Red de Fragilidad y Envejecimiento Saludable (CIBERFES), Instituto de Salud Carlos III, Madrid, Spain.
[5]Centro de Investigación Biomédica en Red de Bioingeniería, Biomateriales y Nanomedicina; (CIBERBBN), Instituto de Salud Carlos III, Madrid, Spain.
[6]Global Brain Health Institute, San Francisco, CA, USA.
[7]Nuclear Medicine Department, Hospital Clínic Barcelona - IDIBAPS, Spain.
[8]F. Hoffmann-La Roche Ltd, Basel, Switzerland.
[9]Department of Psychiatry and Neurochemistry, Institute of Neuroscience & Physiology, the Sahlgrenska Academy at the University of Gothenburg, Mölndal, Sweden[10] Banner Alzheimer's Institute and University of Arizona, Phoenix, AZ, USA
[11] Banner Sun Health Research Institute, Sun City, AZ 85351, USA
[12]Department of Psychiatry, University of Pittsburgh, PA, USA.
[13]Turku PET Centre, University of Turku, Turku, Finland.
[14]Lilly Research Laboratories, Eli Lilly and Company, Indianapolis, IN, USA.
[15]Stark Neurosciences Research Institute, Indiana University School of Medicine, Indianapolis, IN, USA.
[16]Roche Diagnostics GmbH, Penzberg, Germany.
[17]Roche Diagnostics International Ltd, Rotkreuz, Switzerland.
[18]ADx NeuroSciences, Technologiepark 94, Ghent, Belgium.
[19]Novartis Institutes for BioMedical Research, Translational Medicine, Neuroscience, Basel, Switzerland
[20]Department of Psychiatry and Neurochemistry, Institute of Neuroscience and Physiology, University of Gothenburg, Mölndal, Sweden.
[21]Clinical Neurochemistry Laboratory, Sahlgrenska University Hospital, Mölndal, Sweden.
[22]Department of Neurodegenerative Disease, UCL Institute of Neurology, Queen Square, London, United Kingdom.
[23]UK Dementia Research Institute at UCL, London, United Kingdom.
[24]Servei de Neurologia, Hospital del Mar, Barcelona, Spain.
[25]AstraZeneca

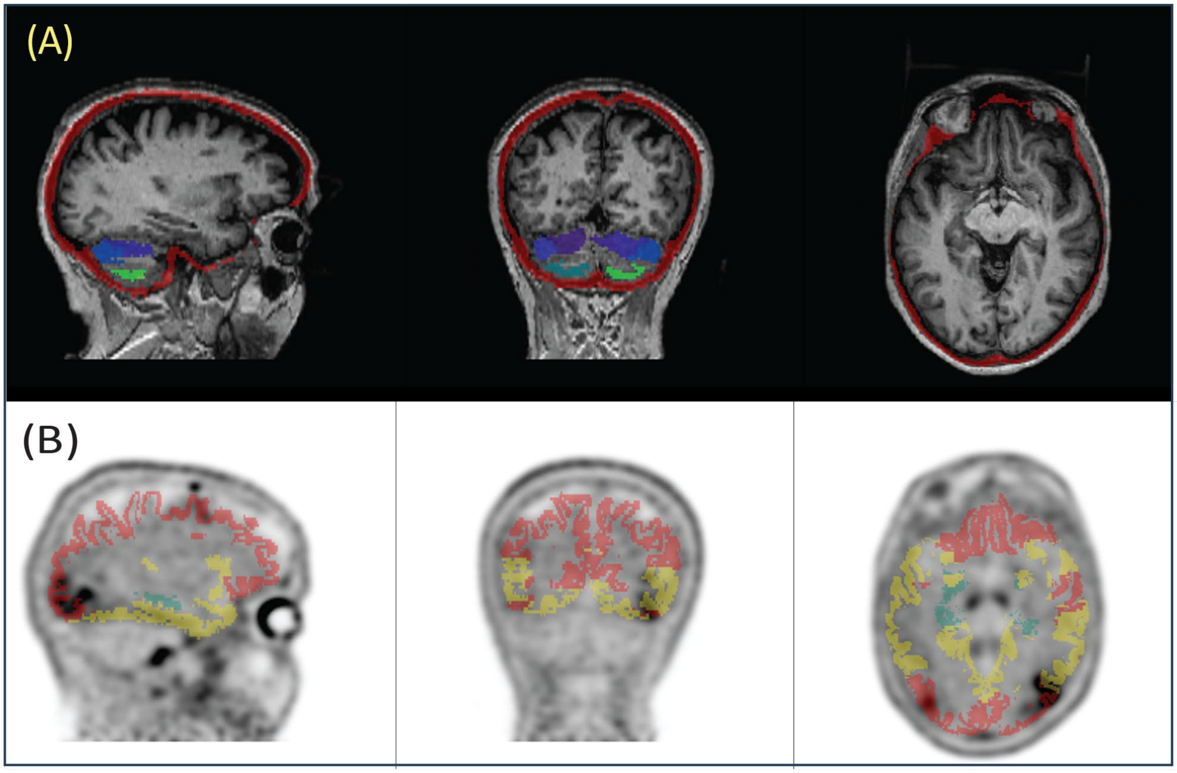

Figure_S1. (A) Fused T1-weighted MRI with meningeal mask and inferior cerebellum as reference region (B) Tau PET scan fused with the subject based cortical ROI, representing different Braak stages.

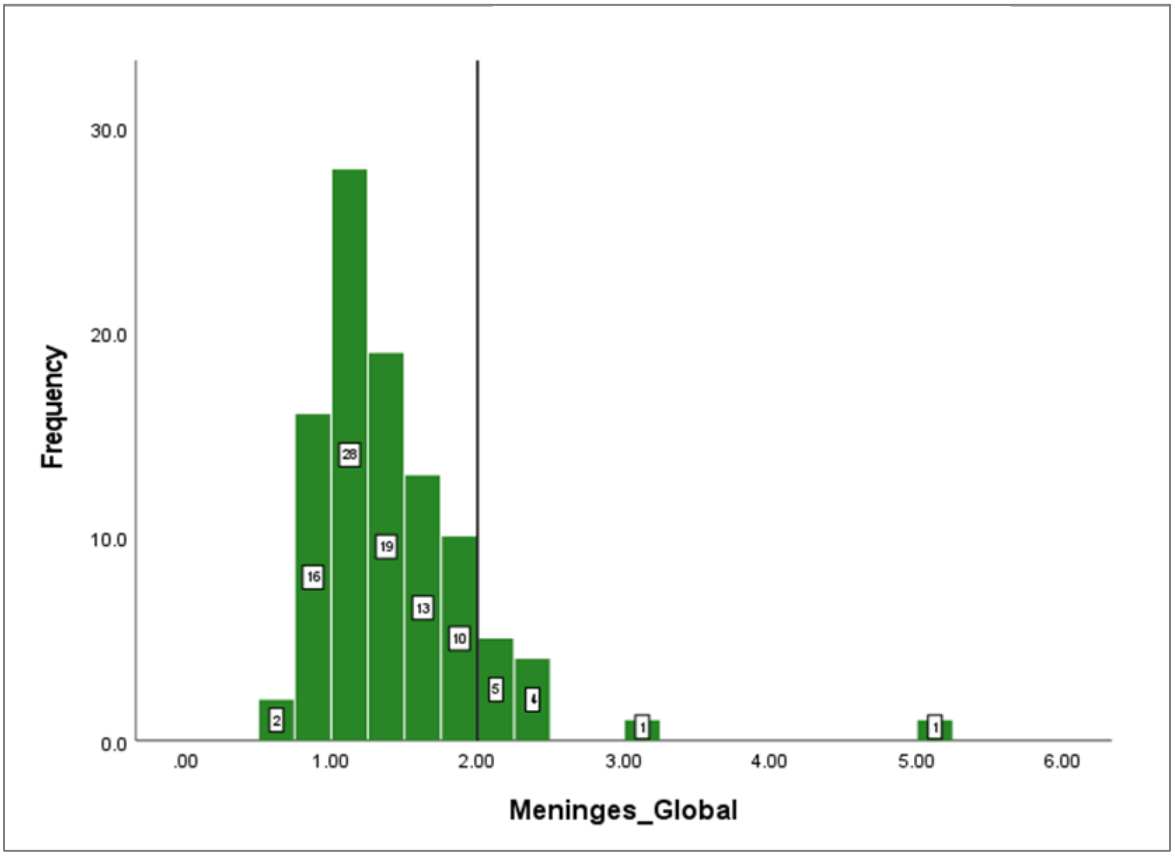

Figure_S2. Histogram of the global meningeal SUVr. It should be noted that 11 participants showed meningeal SUVr above 2.

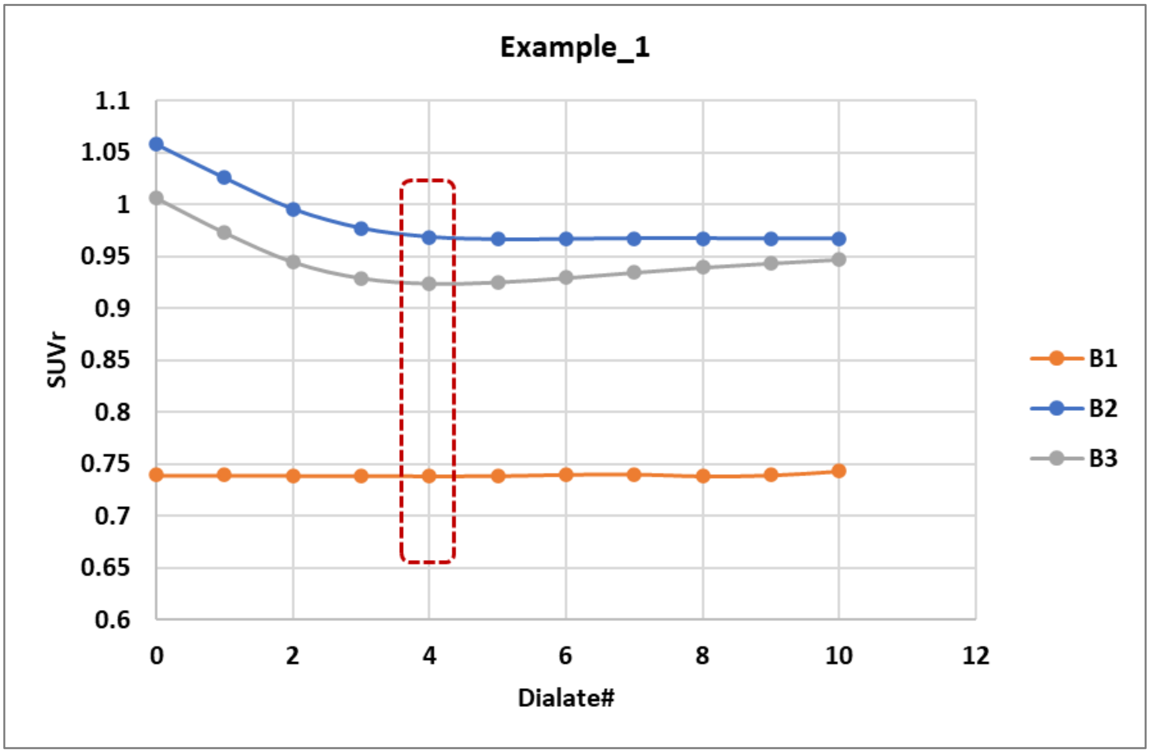

Figure_S3. An example of the procedure of choosing optimal SUVr for the 11 participants with high meningeal uptake. B1, B2, and B3 in the figure refer to Braak I/II, Braak III/IV, and Braak V/VI respectively.

	SUVr (Mean)	SUVr (SD)	Positivity threshold (Mean+2SD)
**Braak I/II**	1.07	0.14	1.35
**Braak III/IV**	1.12	0.10	1.31
**Braak V/VI**	1.06	0.09	1.25
**Temporal MetaROI**	1.15	0.11	1.37
**NEO-T**	1.14	0.10	1.35
**Global**	1.08	0.09	1.27

Table S1. Positivity thresholds for each Braak ROI, the temporal MetaROI, the NEO-T composite, and the global ROI, defined as mean + 2 SD of SUVRs derived from the control group (CSF-based A-T-).

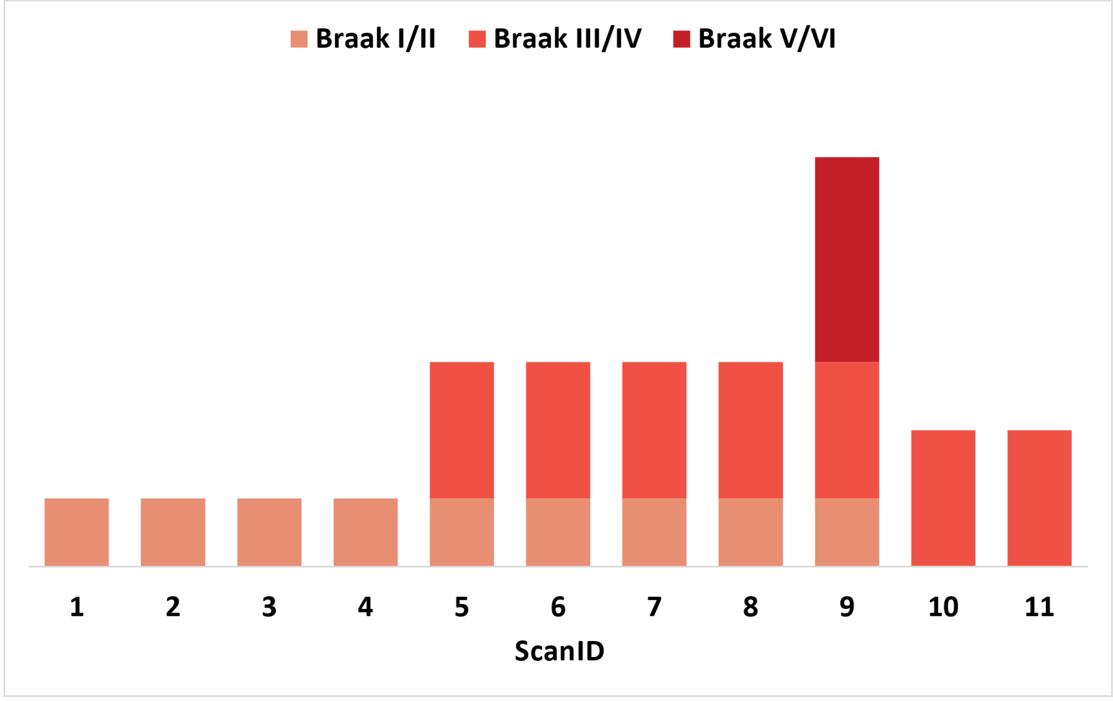

Figure_S4. hierarchal presentation of tau PET positivity for each Braak stage.

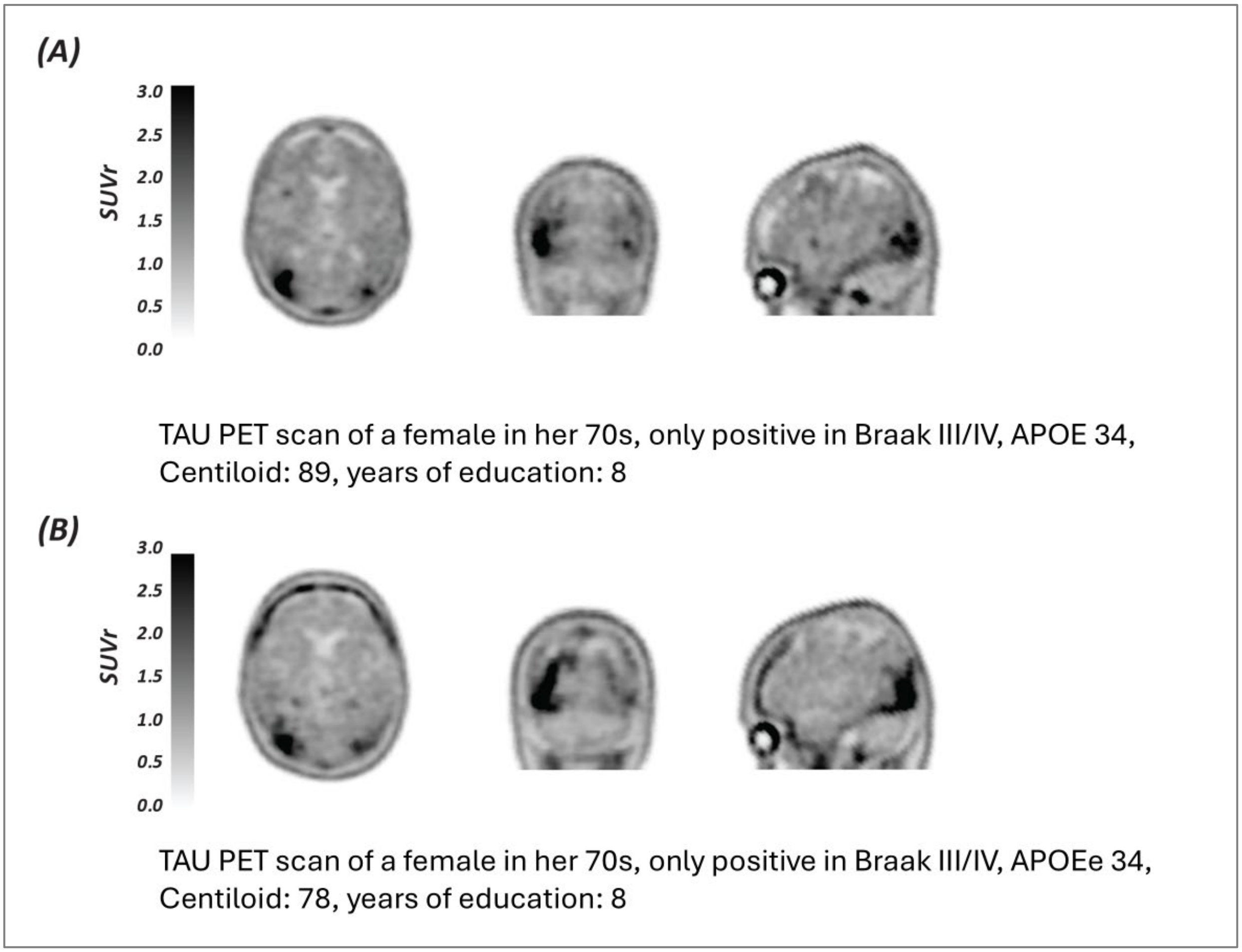

Figure_S5. Visual inspections of the tau PET scans deviating from Braak staging hierarchy, and their corresponding demographical information.

Fluid biomarkers	Partial_Correlation	95%CI	p_value
**Plasma ptau181 (Simoa)**	**0.34**	**0.07-0.60**	**0.001**
**Plasma ptau217 (Lilly)**	**0.29**	**0.04-0.55**	**0.005**
**Plasma ptau231 (Simoa)**	**0.27**	**0.04-0.54**	**0.009**
Plasma ptau181 (RocheNTK)	0.18	-0.01-0.37	n.s
Plasma ptau181/Aβ40 (RocheNTK)	0.17	-0.03-0.39	n.s
Plasma ptau181/Aβ42 (RocheNTK)	0.17	-0.06-0.41	n.s
Plasma Aβ42/40 (RocheNTK)	-0.08	-0.34-0.20	n.s
**CSF ptau217 (Lilly)**	**0.46**	**0.08-0.73**	**<0.0001**
**CSF ptau181/Aβ42 (RocheNTK)**	**0.38**	**0.09-0.62**	**<0.0001**
CSF ptau205 (Simoa)	0.32	-0.03-0.66	n.s
CSF ptau181/Aβ40 (RocheNTK)	0.28	0.00-0.54	n.s
**CSF Aβ42/40 (RocheNTK)**	**0.25**	**0.01-0.46**	**0.03**
CSF NTAtau (Simoa)	0.24	-0.07-0.57	n.s
CSF ptau181 (RocheElecsys)	0.24	-0.04-0.49	n.s
CSF ptau235 (Simoa)	0.23	-0.07-0.54	n.s
CSF ptau181 (RocheNTK)	0.22	-0.05-0.47	n.s
CSF ttau (RocheElecsys)	0.21	-0.05-0.43	n.s
CSF ttau (RocheNTK)	0.19	-0.07-0.40	n.s
CSF Aβ42 (RocheNTK)	-0.14	-0.35-0.09	n.s
CSF Aβ42 (RocheElecsys)	-0.16	-0.35-0.06	n.s

Table_S2. Partial correlations and corresponding 95% confidence intervals between Braak III/IV SUVr and fluid biomarkers, adjusted for age, sex, APOE-ε4 carriership, and the time interval between PET imaging and fluid biomarker measurements.

**Biomarker**	**AUC**	**95%CI**	**P-value**
CSF ptau235 (Simoa)	0.61	0.32-0.89	n.s
Plasma Aβ42/40 (RocheNTK)	0.64	0.48-0.80	n.s
**Plasma ptau231 (Simoa)**	**0.74**	**0.53-0.97**	**0.009**
**Plasma ptau181 (Simoa)**	**0.77**	**0.56-0.98**	**0.003**
**Plasma ptau217 (Lilly)**	**0.77**	**0.59-0.96**	**0.003**
**Plasma ptau181/Aβ42 (RocheNTK)**	**0.79**	**0.65-0.93**	**0.002**
**Plasma ptau181 (RocheNTK)**	**0.79**	**0.65-0.93**	**0.002**
**Plasma ptau181/Aβ40 (RocheNTK)**	**0.80**	**0.66-0.94**	**0.002**
**CSF NTAtau**	**0.84**	**0.72-0.97**	**0.005**
**CSF Aβ42/40 ratio**	**0.88**	**0.77-0.99**	**<0.001**
**Centiloid**	**0.89**	**0.81-0.97**	**<0.0001**
**CSF ptau181/Aβ40**	**0.91**	**0.84-0.98**	**<0.0001**
**CSF ptau205 (Simoa)**	**0.91**	**0.83-1.00**	**<0.0001**
**CSF ptau181/Aβ42**	**0.91**	**0.82-1.00**	**<0.001**
**CSF ptau217 (Lilly)**	**0.96**	**0.91-1.00**	**<0.0001**

Table_S3. Area under curve (AUC; 95%CI), corresponding p-value of core AD fluid biomarkers and Centiloid for predicting Braak I/II positivity, extracted from ROC analysis.

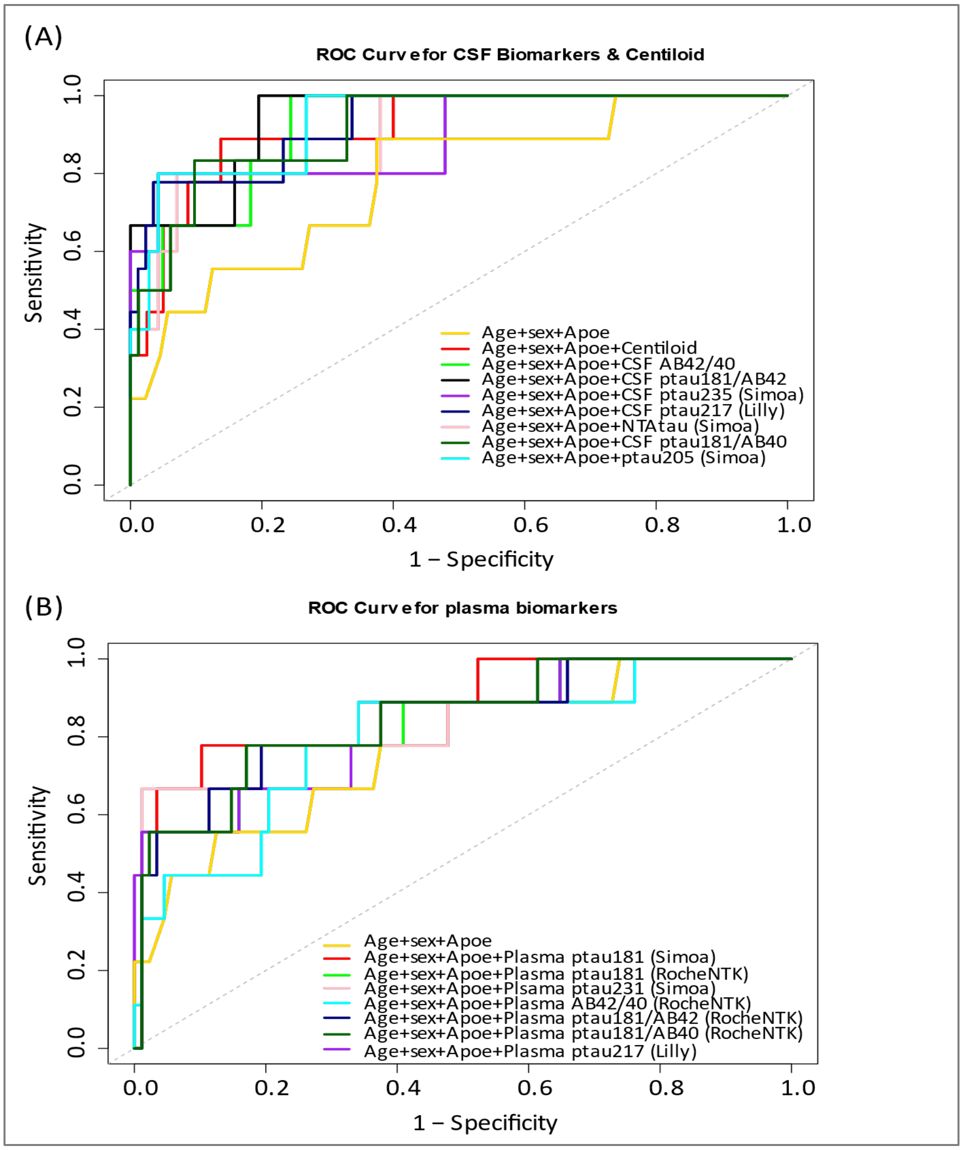

Figure_S6. ROC of the CSF (A) and plasma (B) biomarkers, adjusted for age, sex, and APOE ε4 carriership.

	Total	*A-T-*	*A(Gz)T-*	*A+T-*	*A+T+*
N	99	42	21	17	9
Age, Mean (SD) [53-78 years]	65.15±5.00	63.42±4.18	65.38±5.15	67.06±4.95	69.66±4.58
Sex, N (%) [Female]	57 (57.57%)	23 (54.76%)	11 (52.38%)	13 (76.47%)	4 (44.44%)
APOE-ε4, N (%) [Carriers]	63 (63.63%)	24 (57.14%)	13 (61.90%)	13 (76.47%)	6 (66.66%)
Years of Education	13.35±3.78	14.28±4.12	13.05±3.95	13.48±4.27	13.00±2.23
CSF Aβ42/40, Mean (SD)	0.06±0.02	0.08±0.02	0.05±0.01	0.04±0.006	0.04±0.009
CSF ptau181, Mean (SD), pg/mL	21.92±9.40	16.46±7.15	18.69±7.08	24.05±6.33	33.28±9.03
CSF ptau181/Aβ40	0.001±0.00031	0.0009±0.0001	0.001±0.0002	0.001±0.0002	0.001±0.0003
CSF ptau181/Aβ42	0.02±0.013	0.01±0.006	0.02±0.01	0.03±0.008	0.04±0.01
Centiloid	22.51±26.25	0.68±7.80	23.06±8.50	57.44±14.92	57.24±16.52
CenTauR z-MetaROI	1.09±2.65	0.20±1.51	0.63±1.33	1.47±1.74	6.51±4.95
Hippocampal volume (mm^3)	8860.14±1521.54	8806.12±991.28	8387.34±1050.71	8393.33±1002.85	8495.24±1833.92
Z-Scored PACC, Mean (SD)	0.05±0.62	0.23±0.58	-0.05±0.62	-0.05±0.63	-0.36±0.63

Table_S4. Demographic information, cognition, Centiloid, and CSF biomarkers of the participants stratified by PET-based staging

Plasma Biomarker	Group1	Group2	n1	n2	statistic	p.adj.signif
**AB42/40 (RocheNTK)**	**A-T-**	**A(Gz)T-**	**42**	**21**	**197**	*******
**AB42/40 (RocheNTK)**	**A-T-**	**A+T-**	**42**	**17**	**60**	********
**AB42/40 (RocheNTK)**	**A-T-**	**A+T+**	**42**	**9**	**67**	******
AB42/40 (RocheNTK)	A(Gz)T-	A+T-	21	17	117	ns
AB42/40 (RocheNTK)	A(Gz)T-	A+T+	21	9	92	ns
AB42/40 (RocheNTK)	A+T-	A+T+	17	9	100	ns
Ptau231 (Simoa)	A-T-	A(Gz)T-	42	21	389	ns
**Ptau231 (Simoa)**	**A-T-**	**A+T-**	**42**	**17**	**131**	*******
**Ptau231 (Simoa)**	**A-T-**	**A+T+**	**42**	**9**	**72**	******
**Ptau231 (Simoa)**	**A(Gz)T-**	**A+T-**	**21**	**17**	**96**	*****
Ptau231 (Simoa)	A(Gz)T-	A+T+	21	9	48	ns
Ptau231 (Simoa)	A+T-	A+T+	17	9	62	ns
**Ptau181/AB40 (RocheNTK)**	**A-T-**	**A(Gz)T-**	**42**	**21**	**192**	*******
**Ptau181/AB40 (RocheNTK)**	**A-T-**	**A+T-**	**42**	**17**	**60**	********
**Ptau181/AB40 (RocheNTK)**	**A-T-**	**A+T+**	**42**	**9**	**28**	********
Ptau181/AB40 (RocheNTK)	A(Gz)T-	A+T-	21	17	119	ns
Ptau181/AB40 (RocheNTK)	A(Gz)T-	A+T+	21	9	51	ns
Ptau181/AB40 (RocheNTK)	A+T-	A+T+	17	9	63	ns
**Ptau181 (RocheNTK)**	**A-T-**	**A(Gz)T-**	**42**	**21**	**210**	******
**Ptau181 (RocheNTK)**	**A-T-**	**A+T-**	**42**	**17**	**47.5**	********
**Ptau181 (RocheNTK)**	**A-T-**	**A+T+**	**42**	**9**	**32**	*******
**Ptau181 (RocheNTK)**	**A(Gz)T-**	**A+T-**	**21**	**17**	**83.5**	*****
Ptau181 (RocheNTK)	A(Gz)T-	A+T+	21	9	48	ns
Ptau181 (RocheNTK)	A+T-	A+T+	17	9	74	ns
**Ptau217 (Lilly)**	**A-T-**	**A(Gz)T-**	**42**	**21**	**259**	*****
**Ptau217 (Lilly)**	**A-T-**	**A+T-**	**42**	**17**	**82**	********
**Ptau217 (Lilly)**	**A-T-**	**A+T+**	**42**	**9**	**53**	******
**Ptau217 (Lilly)**	**A(Gz)T-**	**A+T-**	**21**	**17**	**71**	******

**Ptau217 (Lilly)**	**A(Gz)T-**	**A+T+**	**21**	**9**	**44**	*****
Ptau217 (Lilly)	A+T-	A+T+	17	9	67	ns
**Ptau181/AB42 (RocheNTK)**	**A-T-**	**A(Gz)T-**	**42**	**21**	**138**	********
**Ptau181/AB42 (RocheNTK)**	**A-T-**	**A+T-**	**42**	**17**	**27**	********
**Ptau181/AB42 (RocheNTK)**	**A-T-**	**A+T+**	**42**	**9**	**19**	********
Ptau181/AB42 (RocheNTK)	A(Gz)T-	A+T-	21	17	112	ns
Ptau181/AB42 (RocheNTK)	A(Gz)T-	A+T+	21	9	54	ns
Ptau181/AB42 (RocheNTK)	A+T-	A+T+	17	9	70	ns
Ptau181 (Simoa)	A-T-	A(Gz)T-	42	21	308.5	ns
**Ptau181 (Simoa)**	**A-T-**	**A+T-**	**42**	**17**	**103**	*******
**Ptau181 (Simoa)**	**A-T-**	**A+T+**	**42**	**9**	**63.5**	******
**Ptau181 (Simoa)**	**A(Gz)T-**	**A+T-**	**21**	**17**	**90**	*****
**Ptau181 (Simoa)**	**A(Gz)T-**	**A+T+**	**21**	**9**	**44**	*****
Ptau181 (Simoa)	A+T-	A+T+	17	9	57	ns

Table_S5. Pairwise group comparisons of plasma biomarker levels across Amyloid and tau PET stages. The table summarizes statistical comparisons of plasma biomarker concentrations between 4 different groups defined by amyloid (A) and tau (T) PET status: A-T-, A(Gz)T-, A+T-, and A+T+. n1 and n2 refer to the sample sizes in each group. Significance is denoted as follows: $p \leq 0.05$ (), $p \leq 0.01$ (), $p \leq 0.001$ (), and $p \leq 0.0001$ (****); "ns" indicates non-significant results ($p > 0.05$). Stronger group differences are consistently observed for more advanced amyloid and tau pathology stages, particularly between A-T- and A+T+. All analysis done with R, and p-values are adjusted for FDR.

**CSF Biomarkers and Centiloid**	**group1**	**group2**	**n1**	**n2**	**statistic**	**p.adj.signif**
NTAtau (Simoa)	A-T-	A(Gz)T-	34	16	213	ns
**NTAtau (Simoa)**	**A-T-**	**A+T-**	**34**	**13**	**113**	*****
**NTAtau (Simoa)**	**A-T-**	**A+T+**	**34**	**5**	**17**	******
NTAtau (Simoa)	A(Gz)T-	A+T-	16	13	68	ns
**NTAtau (Simoa)**	**A(Gz)T-**	**A+T+**	**16**	**5**	**10**	*****
NTAtau (Simoa)	A+T-	A+T+	13	5	14	ns
Ptau181 (RocheElecsys)	A-T-	A(Gz)T-	41	17	281	ns
**Ptau181 (RocheElecsys)**	**A-T-**	**A+T-**	**41**	**16**	**122**	*******
**Ptau181 (RocheElecsys)**	**A-T-**	**A+T+**	**41**	**6**	**12**	*******
**Ptau181 (RocheElecsys)**	**A(Gz)T-**	**A+T-**	**17**	**16**	**71**	*****
**Ptau181 (RocheElecsys)**	**A(Gz)T-**	**A+T+**	**17**	**6**	**7**	******
Ptau181 (RocheElecsys)	A+T-	A+T+	16	6	24	ns
Ptau181 (RocheNTK)	A-T-	A(Gz)T-	41	17	282	ns
**Ptau181 (RocheNTK)**	**A-T-**	**A+T-**	**41**	**16**	**124**	******
**Ptau181 (RocheNTK)**	**A-T-**	**A+T+**	**41**	**6**	**10**	******
**Ptau181 (RocheNTK)**	**A(Gz)T-**	**A+T-**	**17**	**16**	**69**	*****
**Ptau181 (RocheNTK)**	**A(Gz)T-**	**A+T+**	**17**	**6**	**7**	******
**Ptau181 (RocheNTK)**	**A+T-**	**A+T+**	**16**	**6**	**19**	*****
Ptau205 (Simoa)	A-T-	A(Gz)T-	34	16	192	ns
**Ptau205 (Simoa)**	**A-T-**	**A+T-**	**34**	**13**	**67**	*******
**Ptau205 (Simoa)**	**A-T-**	**A+T+**	**34**	**5**	**4**	*******
Ptau205 (Simoa)	A(Gz)T-	A+T-	16	13	58	ns
**Ptau205 (Simoa)**	**A(Gz)T-**	**A+T+**	**16**	**5**	**5**	******
Ptau205 (Simoa)	A+T-	A+T+	13	5	18	ns
Ptau235 (Simoa)	A-T-	A(Gz)T-	34	16	172	ns
**Ptau235 (Simoa)**	**A-T-**	**A+T-**	**34**	**13**	**65**	*******
**Ptau235 (Simoa)**	**A-T-**	**A+T+**	**34**	**5**	**6**	*******

Ptau235 (Simoa)	A(Gz)T-	A+T-	16	13	58	ns
**Ptau235 (Simoa)**	**A(Gz)T-**	**A+T+**	**16**	**5**	**10**	*****
Ptau235 (Simoa)	A+T-	A+T+	13	5	19	ns
**Ptau217 (Lilly)**	**A-T-**	**A(Gz)T-**	**41**	**17**	**180**	******
**Ptau217 (Lilly)**	**A-T-**	**A+T-**	**41**	**16**	**34**	********
**Ptau217 (Lilly)**	**A-T-**	**A+T+**	**41**	**6**	**2**	********
**Ptau217 (Lilly)**	**A(Gz)T-**	**A+T-**	**17**	**16**	**51**	******
**Ptau217 (Lilly)**	**A(Gz)T-**	**A+T+**	**17**	**6**	**3**	*******
**Ptau217 (Lilly)**	**A+T-**	**A+T+**	**16**	**6**	**11**	******
**AB42/40 (RocheNTK)**	**A-T-**	**A(Gz)T-**	**41**	**17**	**100**	********
**AB42/40 (RocheNTK)**	**A-T-**	**A+T-**	**41**	**16**	**23**	********
**AB42/40 (RocheNTK)**	**A-T-**	**A+T+**	**41**	**6**	**5**	********
AB42/40 (RocheNTK)	A(Gz)T-	A+T-	17	16	83	ns
**AB42/40 (RocheNTK)**	**A(Gz)T-**	**A+T+**	**17**	**6**	**17**	*****
AB42/40 (RocheNTK)	A+T-	A+T+	16	6	27	ns
**Ptau181/AB40 (RocheNTK)**	**A-T-**	**A(Gz)T-**	**41**	**17**	**143**	*******
**Ptau181/AB40 (RocheNTK)**	**A-T-**	**A+T-**	**41**	**16**	**35**	********
**Ptau181/AB40 (RocheNTK)**	**A-T-**	**A+T+**	**41**	**6**	**4**	********
**Ptau181/AB40 (RocheNTK)**	**A(Gz)T-**	**A+T-**	**17**	**16**	**70**	*****
**Ptau181/AB40 (RocheNTK)**	**A(Gz)T-**	**A+T+**	**17**	**6**	**10**	******
**Ptau181/AB40 (RocheNTK)**	**A+T-**	**A+T+**	**16**	**6**	**17**	*****
**Centiloid**	**A-T-**	**A(Gz)T-**	**42**	**21**	**0**	********
**Centiloid**	**A-T-**	**A+T-**	**42**	**17**	**0**	********
**Centiloid**	**A-T-**	**A+T+**	**42**	**9**	**0**	********
**Centiloid**	**A(Gz)T-**	**A+T-**	**21**	**17**	**0**	********
**Centiloid**	**A(Gz)T-**	**A+T+**	**21**	**9**	**10**	********
Centiloid	A+T-	A+T+	17	9	69	ns
**Ptau181/AB42 (RocheNTK)**	**A-T-**	**A(Gz)T-**	**41**	**17**	**83**	********
**Ptau181/AB42 (RocheNTK)**	**A-T-**	**A+T-**	**41**	**16**	**17**	********

**Ptau181/AB42 (RocheNTK)**	**A-T-**	**A+T+**	**41**	**6**	**3**	********
**Ptau181/AB42 (RocheNTK)**	**A(Gz)T-**	**A+T-**	**17**	**16**	**71**	*****
**Ptau181/AB42 (RocheNTK)**	**A(Gz)T-**	**A+T+**	**17**	**6**	**9**	******
**Ptau181/AB42 (RocheNTK)**	**A+T-**	**A+T+**	**16**	**6**	**20**	*****

Table_S6. Pairwise group comparisons of CSF biomarkers levels and Centiloid across Amyloid and tau PET stages. The table summarizes statistical comparisons of CSF biomarker concentrations and Centiloid values between 4 different groups defined by amyloid (A) and tau (T) PET status: A-T-, A(Gz)T-, A+T-, and A+T+. n1 and n2 refer to the sample sizes in each group. Significance is denoted as follows: $p \leq 0.05$ (), $p \leq 0.01$ (), $p \leq 0.001$ (), and $p \leq 0.0001$ (****); "ns" indicates non-significant results ($p > 0.05$). Stronger group differences are consistently observed for more advanced amyloid and tau pathology stages, particularly between A-T- and A+T+. All analysis done with R, and p-values are adjusted for FDR

Cognition	group1	group2	n1	n2	statistic	p.adj.signif
Attention	A-T-	A(Gz)T-	42	21	543	ns
Attention	A-T-	A+T-	42	17	509	ns
Attention	A-T-	A+T+	42	9	243	ns
Attention	A(Gz)T-	A+T-	21	17	214	ns
Attention	A(Gz)T-	A+T+	21	9	99	ns
Attention	A+T-	A+T+	17	9	67	ns
Memory	A-T-	A(Gz)T-	42	21	486	ns
Memory	A-T-	A+T-	42	16	367	ns
Memory	A-T-	A+T+	42	8	223	ns
Memory	A(Gz)T-	A+T-	21	16	165	ns
Memory	A(Gz)T-	A+T+	21	8	106	ns
Memory	A+T-	A+T+	16	8	81	ns
Executive	A-T-	A(Gz)T-	42	21	514	ns
**Executive**	**A-T-**	**A+T-**	**42**	**17**	**546**	*****
Executive	A-T-	A+T+	42	8	204	ns
Executive	A(Gz)T-	A+T-	21	17	246	ns
Executive	A(Gz)T-	A+T+	21	8	90	ns
Executive	A+T-	A+T+	17	8	47	ns
Language	A-T-	A(Gz)T-	42	21	543.5	ns
Language	A-T-	A+T-	42	16	383	ns
Language	A-T-	A+T+	42	8	222.5	ns
Language	A(Gz)T-	A+T-	21	16	155.5	ns
Language	A(Gz)T-	A+T+	21	8	91	ns
Language	A+T-	A+T+	16	8	79	ns

Visual	A-T-	A(Gz)T-	42	21	518	ns
Visual	A-T-	A+T-	42	17	454.5	ns
Visual	A-T-	A+T+	42	9	282.5	ns
Visual	A(Gz)T-	A+T-	21	17	195	ns
Visual	A(Gz)T-	A+T+	21	9	122.5	ns
Visual	A+T-	A+T+	17	9	91	ns
PACC	A-T-	A(Gz)T-	42	21	518	ns
PACC	A-T-	A+T-	42	17	454.5	ns
PACC	A-T-	A+T+	42	9	282.5	ns
PACC	A(Gz)T-	A+T-	21	17	195	ns
PACC	A(Gz)T-	A+T+	21	9	122.5	ns
PACC	A+T-	A+T+	17	9	91	ns

Table_S7. Pairwise group comparisons of attention, memory, executive function, language, visual, and PACC levels across Amyloid and tau PET stages. The table summarizes statistical comparisons of cognitive scores between 4 different groups defined by amyloid (A) and tau (T) PET status: A-T-, A(Gz)T-, A+T-, and A+T+. n1 and n2 refer to the sample sizes in each group. Significance is denoted as follows: $p \leq 0.05$ (), $p \leq 0.01$ (), $p \leq 0.001$ (), and $p \leq 0.0001$ (****); "ns" indicates non-significant results ($p > 0.05$). All analysis done with R, and p-values are adjusted for FDR.

Plasma Biomarker	Group1	Group2	n1	n2	statistic	p.adj.signif
**AB42/40 (RocheNTK)**	**A-T-**	**A(Gz)T-**	**42**	**21**	**685**	*******
**AB42/40 (RocheNTK)**	**A-T-**	**A+T-**	**42**	**17**	**654**	********
**AB42/40 (RocheNTK)**	**A-T-**	**A+T+**	**42**	**9**	**311**	******
AB42/40 (RocheNTK)	A(Gz)T-	A+T-	21	17	240	ns
AB42/40 (RocheNTK)	A(Gz)T-	A+T+	21	9	97	ns
AB42/40 (RocheNTK)	A+T-	A+T+	17	9	53	ns
Ptau231 (Simoa)	A-T-	A(Gz)T-	42	21	389	ns
**Ptau231 (Simoa)**	**A-T-**	**A+T-**	**42**	**17**	**131**	*******
**Ptau231 (Simoa)**	**A-T-**	**A+T+**	**42**	**9**	**72**	******
**Ptau231 (Simoa)**	**A(Gz)T-**	**A+T-**	**21**	**17**	**96**	*****
Ptau231 (Simoa)	A(Gz)T-	A+T+	21	9	48	ns
Ptau231 (Simoa)	A+T-	A+T+	17	9	62	ns
**Ptau181/AB40 (RocheNTK)**	**A-T-**	**A(Gz)T-**	**42**	**21**	**192**	*******
**Ptau181/AB40 (RocheNTK)**	**A-T-**	**A+T-**	**42**	**17**	**60**	********
**Ptau181/AB40 (RocheNTK)**	**A-T-**	**A+T+**	**42**	**9**	**28**	********
Ptau181/AB40 (RocheNTK)	A(Gz)T-	A+T-	21	17	119	ns
Ptau181/AB40 (RocheNTK)	A(Gz)T-	A+T+	21	9	51	ns
Ptau181/AB40 (RocheNTK)	A+T-	A+T+	17	9	63	ns
**Ptau181 (RocheNTK)**	**A-T-**	**A(Gz)T-**	**42**	**21**	**210**	******
**Ptau181 (RocheNTK)**	**A-T-**	**A+T-**	**42**	**17**	**47.5**	********
**Ptau181 (RocheNTK)**	**A-T-**	**A+T+**	**42**	**9**	**32**	*******
**Ptau181 (RocheNTK)**	**A(Gz)T-**	**A+T-**	**21**	**17**	**83.5**	*****
Ptau181 (RocheNTK)	A(Gz)T-	A+T+	21	9	48	ns
Ptau181 (RocheNTK)	A+T-	A+T+	17	9	74	ns
**Ptau217 (Lilly)**	**A-T-**	**A(Gz)T-**	**42**	**21**	**259**	*****
**Ptau217 (Lilly)**	**A-T-**	**A+T-**	**42**	**17**	**82**	********
**Ptau217 (Lilly)**	**A-T-**	**A+T+**	**42**	**9**	**53**	******
**Ptau217 (Lilly)**	**A(Gz)T-**	**A+T-**	**21**	**17**	**71**	******

**Ptau217 (Lilly)**	**A(Gz)T-**	**A+T+**	**21**	**9**	**44**	*****
Ptau217 (Lilly)	A+T-	A+T+	17	9	67	ns
**Ptau181/AB42 (RocheNTK)**	**A-T-**	**A(Gz)T-**	**42**	**21**	**138**	********
**Ptau181/AB42 (RocheNTK)**	**A-T-**	**A+T-**	**42**	**17**	**27**	********
**Ptau181/AB42 (RocheNTK)**	**A-T-**	**A+T+**	**42**	**9**	**19**	********
Ptau181/AB42 (RocheNTK)	A(Gz)T-	A+T-	21	17	112	ns
Ptau181/AB42 (RocheNTK)	A(Gz)T-	A+T+	21	9	54	ns
Ptau181/AB42 (RocheNTK)	A+T-	A+T+	17	9	70	ns
Ptau181 (Simoa)	A-T-	A(Gz)T-	42	21	308.5	ns
**Ptau181 (Simoa)**	**A-T-**	**A+T-**	**42**	**17**	**103**	*******
**Ptau181 (Simoa)**	**A-T-**	**A+T+**	**42**	**9**	**63.5**	******
**Ptau181 (Simoa)**	**A(Gz)T-**	**A+T-**	**21**	**17**	**90**	*****
**Ptau181 (Simoa)**	**A(Gz)T-**	**A+T+**	**21**	**9**	**44**	*****
Ptau181 (Simoa)	A+T-	A+T+	17	9	57	ns

Table_S8. Pairwise group comparisons of the fold of changes for plasma biomarkers across Amyloid and tau PET stages. The table summarizes statistical comparisons of the fold of changes in plasma biomarker concentrations between 4 different groups defined by amyloid (A) and tau (T) PET status: A-T-, A(Gz)T-, A+T-, and A+T+. n1 and n2 refer to the sample sizes in each group. Significance is denoted as follows: $p \leq 0.05$ (), $p \leq 0.01$ (), $p \leq 0.001$ (), and $p \leq 0.0001$ (****); "ns" indicates non-significant results ($p > 0.05$). All analysis done with R, and p-values are adjusted for FDR.

Biomarker	group1	group2	n1	n2	statistic	p.adj.signif
NTAtau (Simoa)	A-T-	A(Gz)T-	34	16	213	ns
**NTAtau (Simoa)**	**A-T-**	**A+T-**	**34**	**13**	**113**	*****
**NTAtau (Simoa)**	**A-T-**	**A+T+**	**34**	**5**	**17**	******
NTAtau (Simoa)	A(Gz)T-	A+T-	16	13	68	ns
**NTAtau (Simoa)**	**A(Gz)T-**	**A+T+**	**16**	**5**	**10**	*****
NTAtau (Simoa)	A+T-	A+T+	13	5	14	ns
Ptau181 (RocheElecsys)	A-T-	A(Gz)T-	41	17	281	ns
**Ptau181 (RocheElecsys)**	**A-T-**	**A+T-**	**41**	**16**	**122**	*******
**Ptau181 (RocheElecsys)**	**A-T-**	**A+T+**	**41**	**6**	**12**	*******
**Ptau181 (RocheElecsys)**	**A(Gz)T-**	**A+T-**	**17**	**16**	**71**	*****
**Ptau181 (RocheElecsys)**	**A(Gz)T-**	**A+T+**	**17**	**6**	**7**	******
Ptau181 (RocheElecsys)	A+T-	A+T+	16	6	24	ns
Ptau181 (RocheNTK)	A-T-	A(Gz)T-	41	17	282	ns
**Ptau181 (RocheNTK)**	**A-T-**	**A+T-**	**41**	**16**	**124**	******
**Ptau181 (RocheNTK)**	**A-T-**	**A+T+**	**41**	**6**	**10**	******
**Ptau181 (RocheNTK)**	**A(Gz)T-**	**A+T-**	**17**	**16**	**69**	*****
**Ptau181 (RocheNTK)**	**A(Gz)T-**	**A+T+**	**17**	**6**	**7**	******
**Ptau181 (RocheNTK)**	**A+T-**	**A+T+**	**16**	**6**	**19**	*****
Ptau205 (Simoa)	A-T-	A(Gz)T-	34	16	192	ns
**Ptau205 (Simoa)**	**A-T-**	**A+T-**	**34**	**13**	**67**	*******
**Ptau205 (Simoa)**	**A-T-**	**A+T+**	**34**	**5**	**4**	*******
Ptau205 (Simoa)	A(Gz)T-	A+T-	16	13	58	ns
**Ptau205 (Simoa)**	**A(Gz)T-**	**A+T+**	**16**	**5**	**5**	******
Ptau205 (Simoa)	A+T-	A+T+	13	5	18	ns
Ptau235 (Simoa)	A-T-	A(Gz)T-	34	16	172	ns
**Ptau235 (Simoa)**	**A-T-**	**A+T-**	**34**	**13**	**65**	*******
**Ptau235 (Simoa)**	**A-T-**	**A+T+**	**34**	**5**	**6**	*******
Ptau235 (Simoa)	A(Gz)T-	A+T-	16	13	58	ns

**Ptau235 (Simoa)**	**A(Gz)T-**	**A+T+**	**16**	**5**	**10**	*****
Ptau235 (Simoa)	A+T-	A+T+	13	5	19	ns
**Ptau217 (Lilly)**	**A-T-**	**A(Gz)T-**	**41**	**17**	**180**	******
**Ptau217 (Lilly)**	**A-T-**	**A+T-**	**41**	**16**	**34**	********
**Ptau217 (Lilly)**	**A-T-**	**A+T+**	**41**	**6**	**2**	********
**Ptau217 (Lilly)**	**A(Gz)T-**	**A+T-**	**17**	**16**	**51**	******
**Ptau217 (Lilly)**	**A(Gz)T-**	**A+T+**	**17**	**6**	**3**	*******
**Ptau217 (Lilly)**	**A+T-**	**A+T+**	**16**	**6**	**11**	******
**AB42/40 (RocheNTK)**	**A-T-**	**A(Gz)T-**	**41**	**17**	**597**	********
**AB42/40 (RocheNTK)**	**A-T-**	**A+T-**	**41**	**16**	**633**	********
**AB42/40 (RocheNTK)**	**A-T-**	**A+T+**	**41**	**6**	**241**	********
AB42/40 (RocheNTK)	A(Gz)T-	A+T-	17	16	189	ns
**AB42/40 (RocheNTK)**	**A(Gz)T-**	**A+T+**	**17**	**6**	**85**	*****
AB42/40 (RocheNTK)	A+T-	A+T+	16	6	69	ns
**Ptau181/AB40 (RocheNTK)**	**A-T-**	**A(Gz)T-**	**41**	**17**	**143**	*******
**Ptau181/AB40 (RocheNTK)**	**A-T-**	**A+T-**	**41**	**16**	**35**	********
**Ptau181/AB40 (RocheNTK)**	**A-T-**	**A+T+**	**41**	**6**	**4**	********
**Ptau181/AB40 (RocheNTK)**	**A(Gz)T-**	**A+T-**	**17**	**16**	**70**	*****
**Ptau181/AB40 (RocheNTK)**	**A(Gz)T-**	**A+T+**	**17**	**6**	**10**	******
**Ptau181/AB40 (RocheNTK)**	**A+T-**	**A+T+**	**16**	**6**	**17**	*****
**Ptau181/AB42 (RocheNTK)**	**A-T-**	**A(Gz)T-**	**41**	**17**	**83**	********
**Ptau181/AB42 (RocheNTK)**	**A-T-**	**A+T-**	**41**	**16**	**17**	********
**Ptau181/AB42 (RocheNTK)**	**A-T-**	**A+T+**	**41**	**6**	**3**	********
**Ptau181/AB42 (RocheNTK)**	**A(Gz)T-**	**A+T-**	**17**	**16**	**71**	*****
**Ptau181/AB42 (RocheNTK)**	**A(Gz)T-**	**A+T+**	**17**	**6**	**9**	******
**Ptau181/AB42 (RocheNTK)**	**A+T-**	**A+T+**	**16**	**6**	**20**	*****

Table_S9. Pairwise group comparisons of the fold of changes for CSF biomarkers across Amyloid and tau PET stages. The table summarizes statistical comparisons of the fold of changes in CSF biomarker concentrations between 4 different groups defined by amyloid (A) and tau (T) PET status: A-T-, A(Gz)T-, A+T-, and A+T+. n1 and n2 refer to the sample sizes

in each group. Significance is denoted as follows: $p \leq 0.05$ (), $p \leq 0.01$ (), $p \leq 0.001$ (), and $p \leq 0.0001$ (****); "ns" indicates non-significant results ($p > 0.05$). All analysis done with R, and p-values are adjusted for FDR.

Reference

1. Milà-Alomà M, Salvadó G, Gispert JD, et al. Amyloid beta, tau, synaptic, neurodegeneration, and glial biomarkers in the preclinical stage of the Alzheimer's continuum. *Alzheimer's & Dementia*. 2020;16(10):1358-1371.
